# *In silico* screening, synthesis, and biological evaluation of pyrazolopyrimidine-derived mTOR inhibitors for anticancer and senomorphic effects

**DOI:** 10.1186/s12935-026-04308-0

**Published:** 2026-05-06

**Authors:** David Rysanek, Zofia Chrienova, Dorota Stary, Marek Bajda, Josef Novak, Pavla Vasicova, Jirina Kroupova, Rudolf Andrys, Adam Skarka, Erika Rousarova, Jan Capek, Tomas Rousar, Zoran Milovanovic, Jelica Grujic-Milanovic, Vesna Jacevic, Jayaprakash N. Kolla, Martin Popr, Dominika Maurencova, Patrik Oleksak, Lukas Fresser, Kamil Kuca, Zdenek Hodny, Eugenie Nepovimova

**Affiliations:** 1 Laboratory of Genome Integrity, Institute of Molecular Genetics of the Czech Academy of Sciences, Videnska, Prague, 1083, 142 00 Czech Republic; 2 Department of Chemistry, Faculty of Science, University of Hradec Kralove, Rokitanskeho 62, Hradec Kralove, 500 03 Czech Republic; 3 Department of Physicochemical Drug Analysis, Faculty of Pharmacy, Jagiellonian University Medical College, Medyczna 9, Krakow, 30-688 Poland; 4 Doctoral School of Medical and Health Sciences, Jagiellonian University Medical College, Medyczna 9, Krakow, 30-688 Poland; 5 Department of Analytical Chemistry, Faculty of Chemical Technology, University of Pardubice, Studentska 573, Pardubice, 532 10 Czech Republic; 6 Department of Biological and Biochemical Sciences, Faculty of Chemical Technology, University of Pardubice, Studentska 573, Pardubice, 532 10 Czech Republic; 7 Special Police Unit, Police Department of the City of Belgrade, Ministry of Interior, Trebevićka 12/A, Belgrade, 11030 Serbia; 8 Institute for Medical Research, Department for Cardiovascular Research, University of Belgrade, National Institute of the Republic of Serbia, Dr Subotića 4, Belgrade, 11029 Serbia; 9 Department of Experimental Toxicology and Pharmacology, National Poison Control Centre, Military Medical Academy, 17 Crnotravska, Belgrade, 11000 Serbia; 10 Department for Pharmacological Science, Medical Faculty of the Military Medical Academy, University of Defence in Belgrade, 17 Crnotravska, Belgrade, 11000 Serbia; 11 CZ-OPENSCREEN: National Infrastructure for Chemical Biology, Institute of Molecular Genetics of the Czech Academy of Sciences, Videnska, Prague 4, 1083, 142 00 Czech Republic; 12 Centre for Basic and Applied Research, Faculty of Informatics and Management, University of Hradec Kralove, Hradec Kralove, 500 03 Czech Republic

**Keywords:** mTOR, Cellular senescence, Cell migration, Aging, Cancer, Torkinib, Senescence-associated secretory phenotype, Inflammation

## Abstract

**Background:**

Cellular senescence is a stress-induced state characterized by irreversible cell cycle arrest. Senescent cells accumulate during aging and contribute to age-related diseases, including neurodegeneration, cancer, and type 2 diabetes mellitus. The mTOR signaling pathway plays a critical role in maintaining and regulating senescence-associated features.

**Methods:**

We employed virtual high-throughput screening and fragment-based design to identify novel small-molecule competitive mTOR kinase inhibitors with favorable physicochemical properties. Six lead compounds (1–6) were selected, and torkinib (7) was synthesized and used as a reference.

**Results:**

Biochemical and cell-based assays revealed that torkinib and compounds 5 and 6 inhibited mTORC1-mediated phosphorylation of p70 S6K. Compound 5 exhibited cytostatic effects in both non-transformed human cells and glioma cancer cells, with greater sensitivity observed in the latter. Unlike the rapalog temsirolimus, both torkinib and compound 5 suppressed migration in multiple glioblastoma cell lines. Notably, compound 5 induced a transient autophagy flux distinct from that elicited by other tested mTOR inhibitors. Furthermore, compound 5 reduced radiation-induced expression of senescence-associated secretory phenotype (SASP) markers, including IL-1α, IL-6, and IL-8. Additional senomorphic effects included decreased cell size and reduced senescence-associated β-galactosidase activity. In vivo, compound 5 showed slightly higher toxicity than torkinib, likely due to improved solubility.

**Conclusions:**

Compound 5 demonstrates distinct biological effects compared to torkinib and represents a promising candidate for further development as an mTOR inhibitor targeting both cancer and senescent cells.

**Supplementary Information:**

The online version contains supplementary material available at 10.1186/s12935-026-04308-0.

## Introduction

In organism, cells undergo temporary or permanent non-cycling or cycling states to maintain tissue and organ integrity. The processes of withdrawing or reentering the cell cycle are highly regulated and determine cell functionality. In cycling cells, growth factors and nutrient supply stimulate growth-promoting pathways such as MEK/MAPK and PI3K/mTOR. These pathways enhance cellular mass growth and induce cyclin D1, which initiates cell cycle progression. In proliferating cells, mass growth is balanced by cell division [1]. In absence of growth stimulation (e.g., serum withdrawal, lack of growth factors and nutrients), MAPK and mTOR are deactivated. Additionally, an increase in cell cycle inhibitory proteins (e.g., cyclin-dependent kinases; CDK) halts the cell division cycle and blocks proliferation. The reversibility of this non-proliferating state is defined as quiescence, and the ability to resume proliferation, for example, by adding growth stimulators, distinguishes quiescent cells from terminally differentiated or senescent cells [2]. On the other hand, senescence is associated with permanent cell cycle arrest [3]. The cell cycle can be directly blocked by the induction of protein inhibitors of CDKs, such as p21^waf1^ (p21) and p16^INK4A^ (p16), which are inducible by numerous stressors. When the cell cycle is blocked in this manner, mTOR and MAPK remain fully activated, resulting in unbalanced growth without initiating cell division and inducing cyclin D1 [2, 3].

Senescent cells secrete a wide range of bioactive factors, including inflammatory cytokines, chemokines, growth factors, tissue morphogens, extracellular matrix components and modifiers, lipids, nucleotides, either as extracellular vesicles or soluble factors. Collectively, these are termed the senescence-associated secretory phenotype (SASP) [4]. The SASP can have both beneficial and harmful consequences depending on the context. Through SASP, senescent cells can alter tissue microenvironments, attract immune cells, and induce malignant phenotypes in nearby cells. Conversely, a localized, time-limited SASP may be necessary for resolving tissue damage, particularly in younger individuals. The SASP can alert nearby cells to potential danger and promote the immune clearance of the damaged cells [5]. Senescent cells accumulate in the organism during normal aging, limiting lifespan and promoting age-related pathologies such as neurodegenerative disorders, cancer, and type 2 diabetes mellitus [6].

Evidence indicates that mTOR-dependent signaling is essential for maintaining or implementing various aspects of cellular senescence. Depending on the cell type and biological context, inhibiting mTOR in senescent cells can reverse senescence, induce quiescence or cell death, or exacerbate some features of senescent cells while inhibiting others [7]. The mechanistic target of rapamycin (mTOR) can assemble with different proteins to form two types of polyprotein complexes: mTOR complex 1 (mTORC1) and mTOR complex 2 (mTORC2) [8]. mTORC1 is a crucial regulator of cell growth and, according to early studies, is the only complex sensitive to rapamycin [6, 9]. However, subsequent research has shown that prolonged rapamycin treatment can inhibit mTORC2 assembly and function in some cell lines [10]. mTORC2 is involved in metabolic processes, immune regulation, and actin cytoskeleton regulation. The exact role of mTORC2 in aging remains unclear, as longevity studies have produced controversial results 6].

The mTOR kinase domain consists of approximately 550 residues and includes a smaller *N*-terminal lobe and a larger *C*-terminal lobe. The *N*-lobe contains the FKBP12-rapamycin binding (FRB) domain, which is necessary for binding the rapamycin complex with the large FK506-binding protein (FKBP12). The FRB domain is proposed to act as a gatekeeper, reducing the accessibility of the catalytic cleft [11, 12]. The kinase active site is located in the catalytic cleft between the *N*-lobe and *C*-lobe and is formed by a conserved adenosine triphosphate (ATP)-binding pocket and a catalytic loop. Within the ATP-binding pocket, an ATP molecule interacts with the kinase via hydrophobic interactions of adenine moiety, while the phosphates are stabilized by polar interactions [12, 13].

**Fig. 1:**
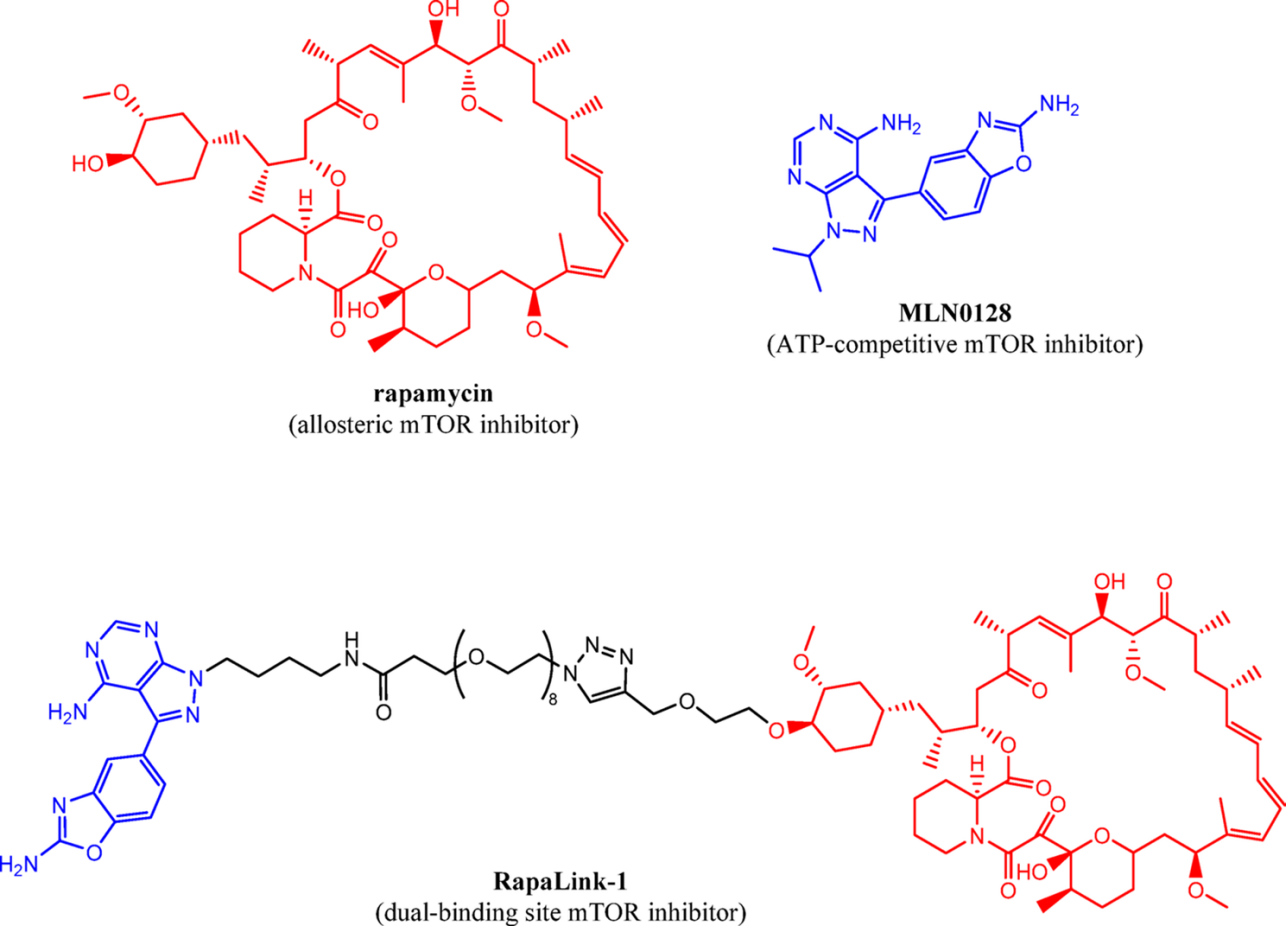
Chemical structures of known mTOR inhibitors

Rapamycin (Fig. 1) was the first compound discovered to inhibit mTOR. Although originally used as an immunosuppressive agent, rapamycin has poor solubility and insufficient bioavailability, which complicate its pharmacological effect. The mechanism of mTOR inhibition involves rapamycin´s interaction with FKBP12, forming a triple complex with the FRB domain of mTOR. This allosteric modification alters the spatial arrangement of the protein active site, ultimately blocking its kinase activity [14]. ATP-competitive mTOR inhibitors directly target the ATP-binding site of the mTOR kinase catalytic domain. These compounds generally show poor selectivity between the mTORC1 and mTORC2. ATP-competitive mTOR inhibitors are a major focus in anti-cancer drug development [15]. Due to challenges with drug resistance in novel mTOR inhibitor design, a new drug combination concept has been presented to address these issues. The ATP-competitive inhibitor MLN0128 (Fig. 1) and rapamycin were combined with an inert linker to form a compound named RapaLink-1 (Fig. 1), which exhibits significant inhibitory properties on both rapamycin-binding and ATP-binding site mutants [16].

Inhibiting the mTOR signaling pathway is an intriguing therapeutic strategy for various age-related diseases, particularly those characterized by extensive cellular proliferation (such as cancer) or senescence (e.g., Alzheimer´s disease). Although existing mTOR inhibitors have limitations, including issues of efficacy and specificity, the search for novel inhibitors continues.

## Design

Previously, we investigated the pharmacologically significant binding sites of mTOR, specifically the FRB domain and the catalytic active site, and validated docking protocols for discovering novel mTOR kinase modulators [17]. The contribution presents a follow-up study to our earlier research, in which we selected potential small molecules from the obtained hits and tested them for their anti-proliferative, senolytic, and senomorphic activity, as well as mTOR inhibition [18]. As in the previous study, we searched for novel small molecule mTOR kinase inhibitors with suitable physicochemical properties using two different in silico methods. The first method involved virtual high-throughput screening of commercially available compounds from the ZINC database. The second method employed fragment-based design (FBD). For our purposes, we targeted the ATP-binding site. The subsequent selection process focused on lead compounds that were either purchasable or feasible to synthesize (Table 1). In the biological evaluation, torkinib (compound 7) was also synthesized and used as a positive control due to its previously published mTOR inhibitory potential [19].

The anti-aging field represents a newer application for mTOR inhibitors; most of which have been developed to treat cancer. Therefore, we decided to evaluate the lead compounds (1–7) for their anticancer potential as well.

**Table 1:** Lead compounds selected for further analysis

Compound	Chemical structure	Binding site	Access	Method
**1**		ATP	Purchased	ZINC
**2**		ATP	Purchased	ZINC
**3**		ATP	Purchased	ZINC
**4**		ATP	Purchased	ZINC
**5**		ATP	Synthesized	FBD
**6**		ATP	Synthesized	FBD
**7** (torkinib)		ATP	Synthesized	FBD

## Results and discussion

### Computational study

**Fig. 2:**
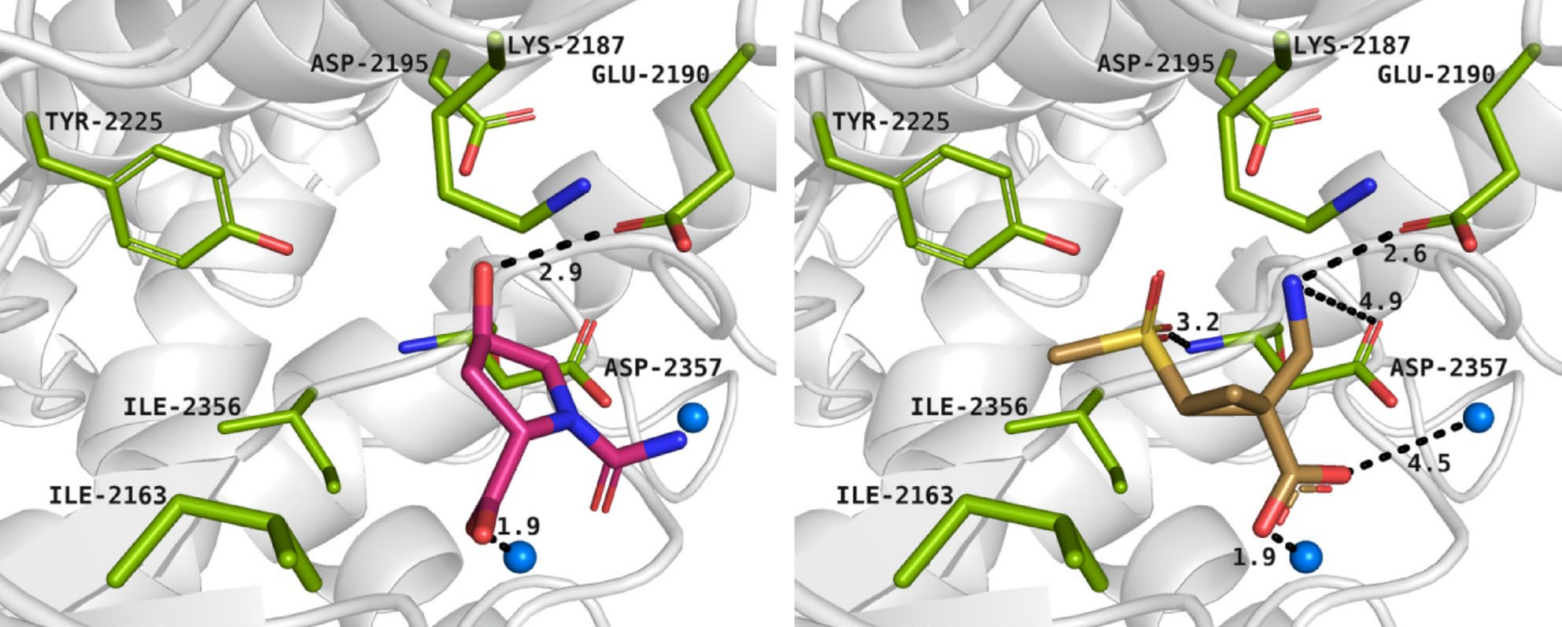
Binding modes of compound 2 (left panel) and compound 4 (right panel) within the ATP-binding site of mTOR kinase. Protein residues are shown in green. Magnesium ions are represented as blue spheres and interactions are indicated by black dashed lines. Compound 2 is shown in pink and compound 4 in brown

Virtual screening of the ZINC database allowed us to select a series of small molecule compounds characterized by a molecular weight of around 200 Da and high solubility. Here, we describe compounds 1–4, which exhibited interesting arrangements in the ATP binding site of mTOR kinase and achieved favorable docking score values ranging from − 4 to -6 kcal/mol, comparable to that of the reference compound 7. Compounds 1–4 were predicted to occupy the phosphate group binding site of ATP. Figure 2 (left panel) shows the binding mode of compound 2, which achieved the most favorable docking score. This compound interacted within the ATP binding site through an ionic bond between the carboxyl group and magnesium ion. Additionally, a hydrogen bond was observed between the oxygen atom of a carbonyl group and Glu2190. Figure 2 (right panel) illustrates compound 4, which revealed several specific interactions within mTOR kinase. The ligand possibly interacted with both magnesium ions, as well as with the ionized amine group forming interaction with carboxyl groups of Glu2 190 and Asp2357. Furthermore, the oxygen atom from the sulfone group was predicted to form a hydrogen bond with the main chain of Asp2357. These observations suggest that compounds 1–4 may serve as fragments for the design of more branched-structure ligands, capable of interacting with residues such as Val2240, Trp2239, and Lys2187, which are important for binding of reference compounds.

**Fig. 3:**
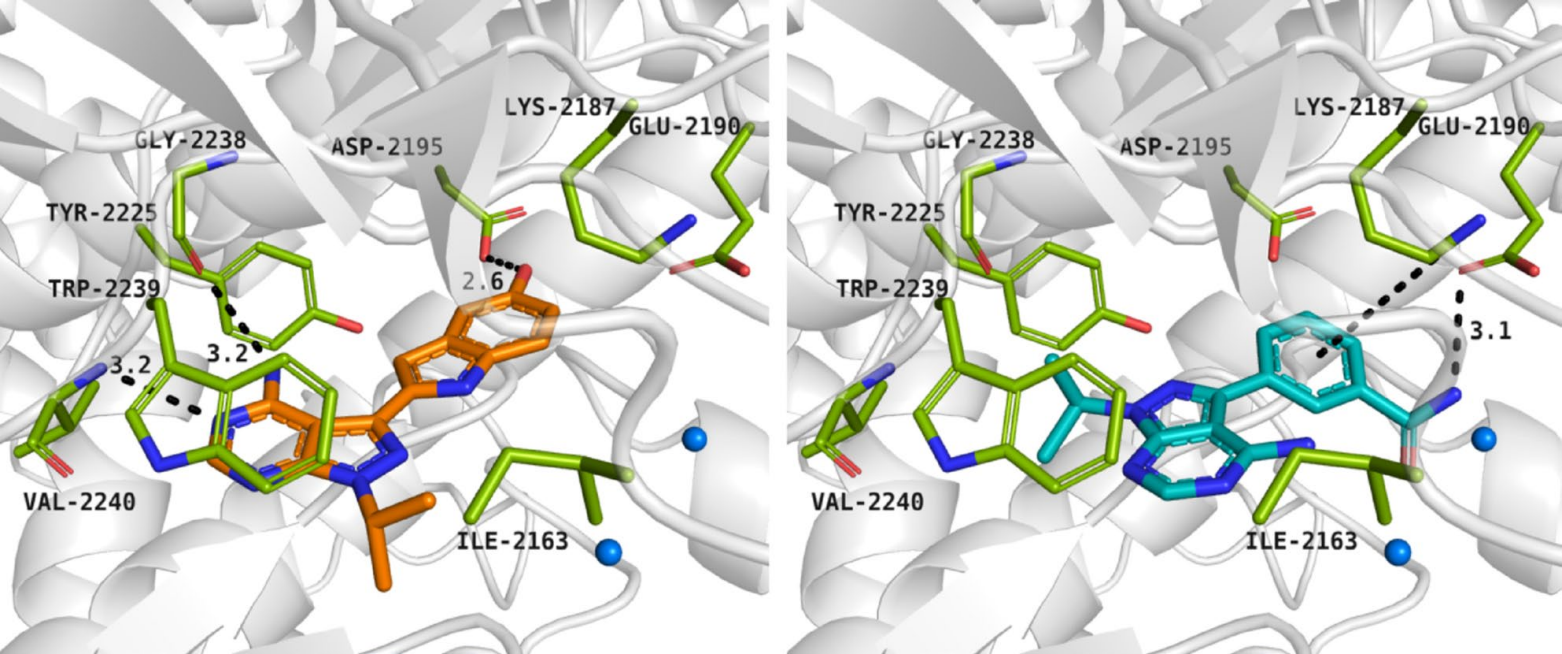
Binding modes of compound 7 (left panel) and compound 5 (right panel) within the ATP-binding site of mTOR kinase. Protein residues are shown in green. Magnesium ions are represented as blue spheres and interactions are indicated by black dashed lines. Compound 7 is shown in orange and compound 5 in teal

Based on the structural fragments, we designed compound 5, which is related to torkinib (7) and its precursor (6). Torkinib (PP 242), a well-known competitive mTOR kinase inhibitor, achieved a docking score of -8.1 kcal/mol. In our previous studies, torkinib was also used to validate the docking procedure. Our approach successfully reproduced the inhibitor binding mode observed in the complex from the Protein Data Bank, supporting the utility of the procedure [17]. Fig. 3 (left panel) shows the binding mode of torkinib in the mTOR binding site, where the pyrazolopyrimidine scaffold mimics the adenine moiety of ATP. Inhibitor forms hydrogen bonds with the main chains of Val2240 and Gly2238. Additionally, the hydroxy group at position 5 of the indole moiety could interact with Asp2195 via a hydrogen bond.

In the case of original compound 5 (Fig. 3, right panel), a different predicted binding mode was observed. The pyrazolopyrimidine moiety with an isopropyl substituent adopted a flipped arrangement compared to the same fragment in torkinib. Aliphatic moiety was located close to Val2240, Gly2238, and Trp2239. The aromatic ring of the benzamide fragment possibly interacted with Lys2187 via cation – *π* interaction. Additionally, the amide group was oriented toward Glu2190, enabling the formation of a hydrogen bond. Overall, compound 5 may represent a promising lead-like structure for further optimization. The proposed binding modes are based on docking results and require experimental confirmation.

### Chemistry

**Scheme 1:**
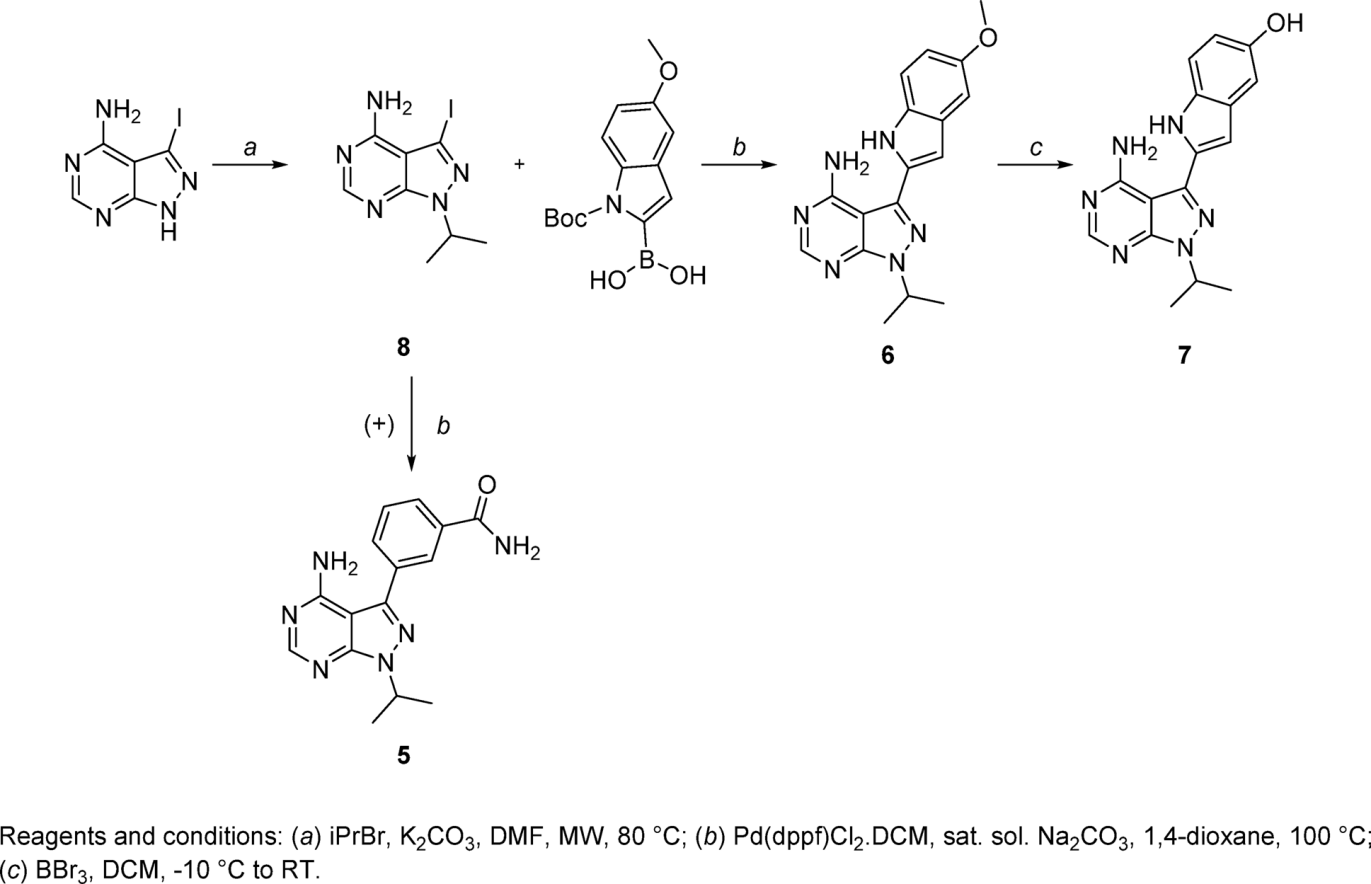
Chemical synthesis of compounds 5, 6, and 7

Compound 7, used as the reference compound, was prepared by the optimized synthetic method described earlier [19, 20]. In the first step, 3-iodo-1*H*-pyrazolo[3,4-*d*]pyrimidin-4-amine was reacted with isopropyl bromide and K_2_CO_3_ in DMF to provide intermediate 8 (Scheme 1a) that was used for further synthesis of the target compounds 5, 6, and 7. In the case of 5 (Scheme 1b), intermediate 8 was left to react with (3-carbamoylphenyl)boronic acid, Pd(dppf)Cl_2_.DCM and Na_2_CO_3_ in 1,4-dioxane by the Suzuki coupling. The exact mechanism was also applied for compound 6, using (1-(*tert*-butoxycarbonyl)-5-methoxy-1*H*-indol-2-yl)boronic acid and Pd(dppf)Cl_2_.DCM catalysis in 1,4-dioxane without further *N*-Boc deprotection. The final demethylation of 6 with boron tribromide afforded the final product 7 (Scheme 1c).

### Physicochemical properties determination

The disadvantage of rapamycin and several other rapalogs lies in their poor pharmacokinetic properties. In addition to favorable activity, the design and synthesis of new potential mTOR inhibitors aim for appropriate physicochemical properties, which are crucial for optimizing absorption, distribution, metabolism, and elimination (ADME) attributes. To objectively evaluate our compounds for their physicochemical attributes, we used the central nervous system multiparameter optimization (CNS MPO) method which assesses six parameters: (a) lipophilicity (logP); (b) distribution coefficient at pH 7.4 (logD); (c) most basic center (pKa); (d) molecular weight (Mw); (e) number of hydrogen-bond donors (HBDs); and (f) topological polar surface area (tPSA). We chose the CNS MPO method to predict the ability of tested compounds (1–7) to permeate across the blood-brain barrier (BBB). Since the BBB is one of the most challenging barriers in the body to cross, we presume that if the studied compounds can overcome this “obstacle” by passive diffusion, their presence in all the body compartments will be all the more likely.

Experimental pKa values were acquired by spectrophotometric titration of compounds containing chromophores close to the ionization center (Table 2). The pKa values for studied compounds ranged from 3.59 to 10.37. Experimental logP and logD values were determined by octanol-aqueous phase extraction and subsequent concentration measurement by LC-MS. LogP and logD values ranged from 1.54 to 3.20 and from 1.57 to 3.31, respectively. Molecular weight, topological polar surface area, and the number of hydrogen-bond donors were calculated using ACDLAbs PhysChemSuite software. The obtained values ranged from 174.15 to 322.36 for Mw, 94.64 to 134.52 for tPSA, and 3 to 4 for HBDs. Collected experimental data (most basic pKa, logP, and logD) and calculated values (tPSA, HBDs, and Mw) were used to calculate the CNS MPO desirability score, as described by Wager et al. [21]. This scoring tool was developed based on ranges of desirable property space and linear transformation functions, yielding values within the range of 0 to 1, designated as T0 for each property. Each parameter was given equal weighting, resulting in a collective score from 0 to 6, with a score exceeding 4 indicating the most desirable outcome [22]. The CNS MPO score of our compounds ranged from 3.1 to 4.3. Therefore, compounds 5 and 6 had desirable values higher than 4. Combined with acquired in vitro data, calculated CNS MPO values were considered an important factor in selecting compound(s) for further in vivo experiments.

**Table 2:** Experimental and calculated physical-chemical properties

Name	Mw	pKa_1_	pKa_2_	logP	logD	tPSA	HBD	MPO
**1**	188.18	n/a	n/a	n/a	n/a	103.86	4	**n/a**
**2**	174.15	n/a	n/a	n/a	n/a	103.86	4	**n/a**
**3**	191.17	3.59 ± 0.03	9.86 ± 0.19	1.10 ± 0.04	1.47 ± 0.14	134.52	4	**3.1**
**4**	229.68	n/a	n/a	1.54 ± 0.04	1.57 ± 0.01	105.84	3	**3.6** ^***a***^
**5**	296.33	4.02 ± 0.02	-	1.52 ± 0.02	1.51 ± 0.01	112.71	4	**4.2**
**6**	322.36	3.95 ± 0.03	-	3.20 ± 0.09	3.31 ± 0.05	94.64	3	**4.3**
**7**	308.34	3.96 ± 0.02	10.37 ± 0.18	2.65 ± 0.02	2.75 ± 0.06	105.64	4	**3.1**
**rapamycin**	914.17	10.7 ± 0.21	-	3.97 ± 0.03	4.63 ± 0.04	195.43	3	**0.7**

^*a*^CNS MPO score for compounds with unknown experimental pKa was calculated with T0(pKa) equal to zero

Finally, solubility in water and buffer at pH 7.4 was assessed for complex characterization using a nephelometry assay (Table 3). The values obtained ranged from poor to excellent solubility. Compound 7 and rapamycin were poorly soluble in water and buffer, with values lower than 6 *µ*g/mL and with average logS_H2O_ and logS_pH7.4_ lower than (-4.95). Compounds 5 and 6 also exhibited low solubility, with average logS_H2O_ and logS_pH7.4_ (-4.01) and (-4.00), respectively. In contrast, compounds 1, 2, 3, and 4 showed solubility higher than 1000 *µ*g/mL, with average logS_H2O_ and logS_pH7.4_ values greater than (-2.29).

**Table 3:** Solubility in water and buffer (pH 7.4)

Name	Mw	Solubility _(H2O)_ [µg/mL]	logS_H2O_	Solubility _(buffer 7.4)_ [µg/mL]	logS_pH 7.4_
**1**	188.18	> 1000	>-2.28	> 1000	>-2.28
**2**	174.15	> 1000	>-2.24	> 1000	>-2.24
**3**	191.17	> 1000	>-2.28	> 1000	>-2.28
**4**	229.68	> 1000	>-2.36	> 1000	>-2.36
**5**	296.33	26.95	-4.04	30.99	-3.98
**6**	322.36	33.45	-3.98	31.48	-4.01
**7**	308.34	< 6	<-4.71	< 6	<-4.71
**rapamycin**	914.17	< 6	<-5.18	< 6	<-5.18

### Biological evaluation

#### In vitro screening and quantification of mTOR inhibition

**Fig. 4:**
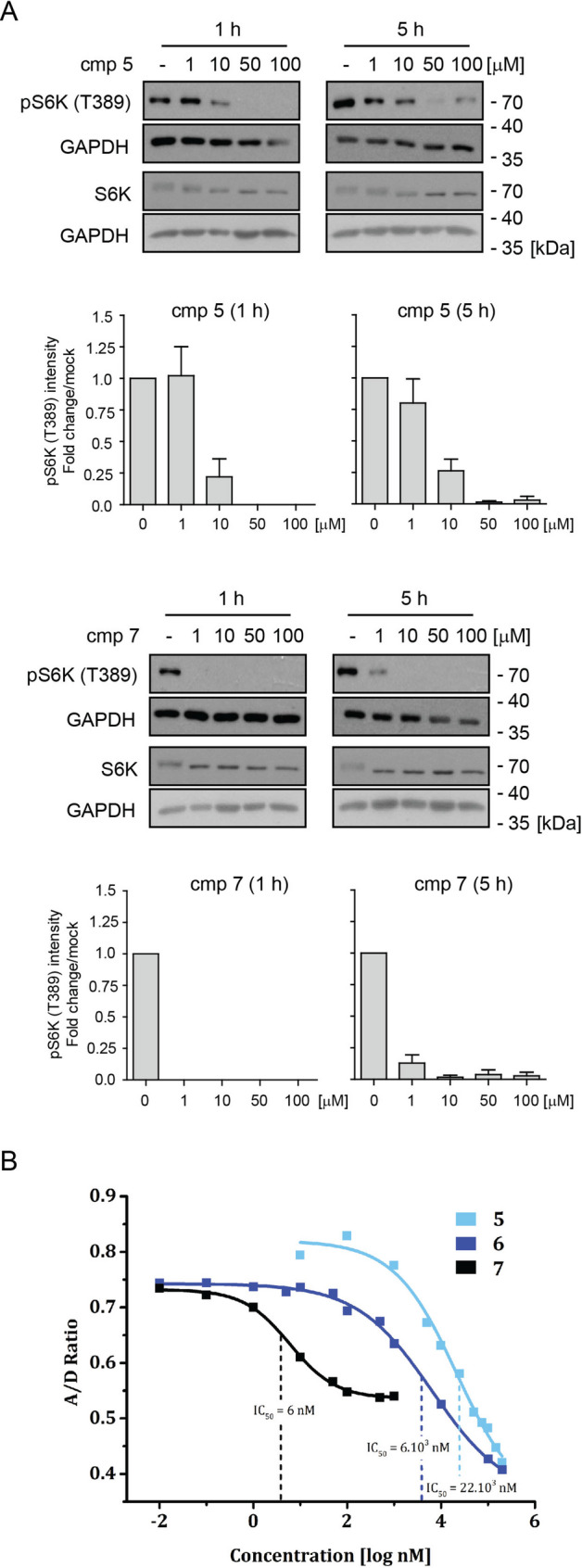
Dose-response inhibition of mTORC1 kinase by compounds 5, 6, and 7. (**A**) BJ cells were exposed to a 1, 10, 50, and 100 *µ*M concentration of compounds 5 and 7 for 1 and 5 h. The levels of p70 S6 kinase (S6K) threonine 389 (Thr389) phosphorylation, mediated by mTORC1, and S6K total level, were detected by immunoblotting using specific antibodies. GAPDH was used as a loading control. The ImageJ 1.48v program was used for quantitative analysis of immunoblots with GAPDH as a loading control. The experiment was done in triplicate. (**B**) Dose-response curves of mTOR inhibition in compounds 5, 6, and 7 (0–200 *µ*M). The mTOR inhibitory activity of compounds 5, 6, and 7 was assessed using a commercial assay employing advanced time-resolved fluorescence resonance energy transfer (TR-FRET) technology. IC_50_ values were calculated using non-linear regression

To assess whether the compounds exhibit the desired biological efficacy, compounds 1–6 were screened for their mTORC1 inhibitory activity by determining the phosphorylation level of the mTORC1 substrate p70 S6K using SDS-PAGE and immunoblotting with specific antibodies. Compound **7** (torkinib) was used as a positive control. Human normal dermal fibroblasts BJ, selected for their optimal response to mTOR inhibition (see ref. [17]), were exposed to a 100 *µ*M concentration of each compound for 1 and 5 h, then harvested for immunoblotting. Compounds 1–4 did not affect p70 S6K phosphorylation. In contrast, compounds 5 and 6 suppressed the phosphorylation status and electrophoretic mobility of p70 S6K after 1 and 5 h (Supplementary Figure S1A). Therefore, compounds 5, 6, and 7 were retested at a lower concentration of 12.5 *µ*M. Compound 7 exhibited the most suppressive effect on p70 S6K phosphorylation, while compounds 5 and 6 showed less pronounced but distinct inhibition (Supplementary Figure S1B). Finally, compounds 5 and 7 were tested to inhibit p70 S6K phosphorylation in concentration range from 1 to 100 *µ*M after 1 and 5 h (Fig. 4A). Compound 5 exhibited a weaker inhibition effect compared to compound 7 (torkinib).

Based on the results presented above, compounds 5 and 6 were selected for further evaluation of their potential to inhibit mTOR protein using a commercial assay with the advanced time-resolved fluorescence resonance energy transfer (TR-FRET) method (Fig. 4B). Compound 7 (torkinib) was used as a positive control. Experimental results are presented as IC_50_ values (the concentration that reduces mTOR activity by 50%). For compound 7, the IC_50_ is 6 nM; for compound 6, it is higher than 1 *µ*M; and for compound 5, it is higher than 10 *µ*M.

In conclusion, the compounds 1–4 showed no effect on inhibition of phosphorylation of p70 S6K. The results obtained for compound 7 are in strong agreement with the study of Apsel et al., reporting inhibition of mTOR at 8 nM [23]. In the case of compound 6, substituting the phenolic group with a methoxy group led to a significant decrease (up to three orders of magnitude) in inhibitory activity against mTOR. As shown in Fig. 4B, compound 5 was the weakest mTOR inhibitor, with an IC_50_ value higher than 10 *µ*M.

#### Cell growth effects of novel compounds

**Fig. 5:**
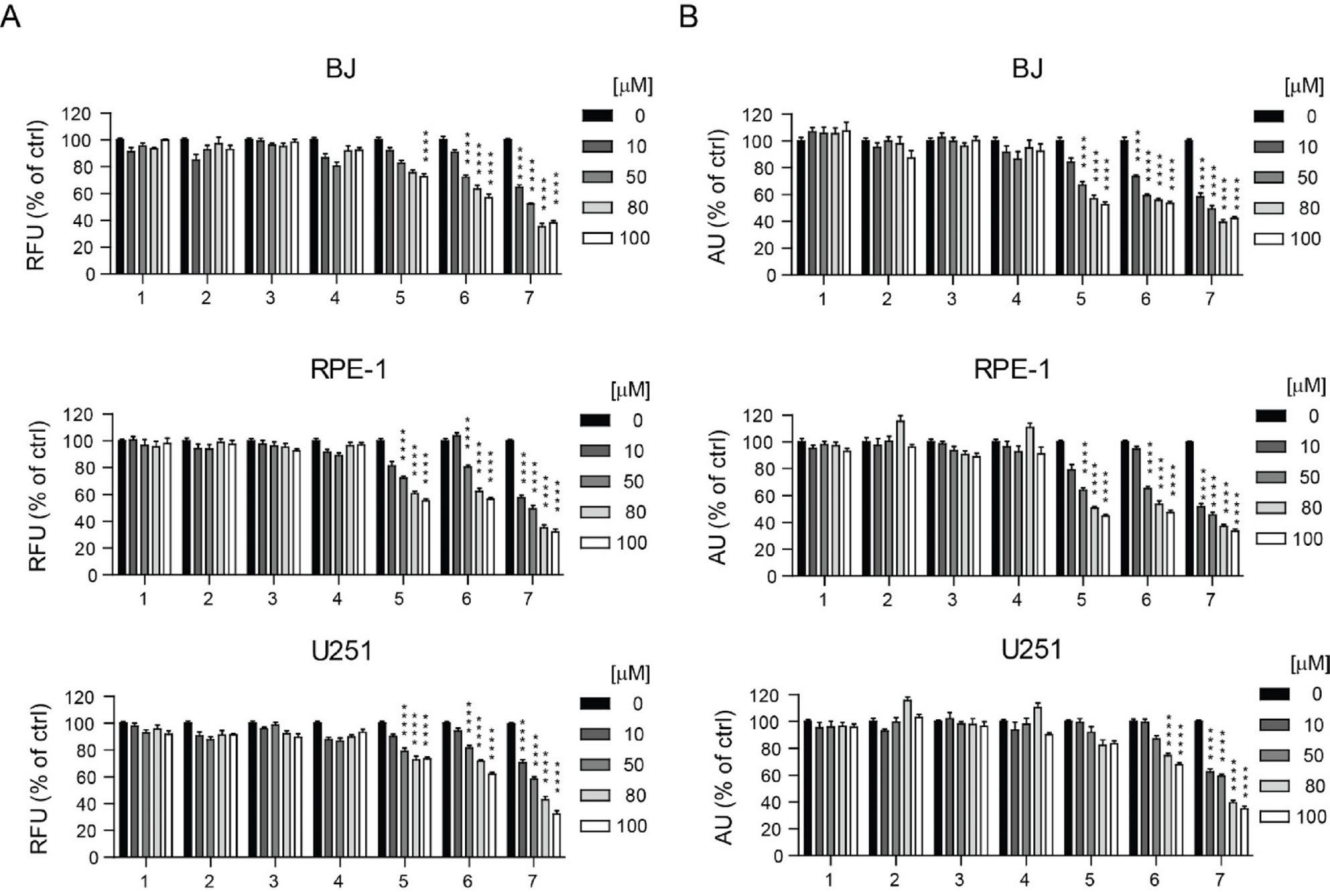
The antiproliferative effects of compounds 1–7. Human BJ fibroblasts, immortalized retinal pigment epithelium RPE-1, and glioma U251 cells were exposed to compounds 1–7 in a concentration range of 0–100 *µ*M for 24 h. Metabolic activity and cell density were determined by (**A**) resazurin and (**B**) crystal violet assays, respectively. The experiment was done in triplicate. Data were normalized to those of untreated cells and plotted as mean ± SEM. Statistical significance was derived using a two-tailed Student’s *t*-test. p-values ^****^p < 0.0001; ^***^p < 0.001; ^**^p < 0.01; *p < 0.05. Only ^****^p < 0.0001 is shown

Further, we assessed the effects of compounds 1–7 on the proliferation of human normal (BJ), immortalized (RPE-1), and cancerous (glioma U251) cells using ‘viability’ tests (resazurin and crystal violet assay, CV). Figure 5 shows that compounds 1–4, consistent with the absence of mTORC1 inhibitory activity, did not affect the growth of any cell type when used at concentrations ranging from 10 to 100 *µ*M for 24 h in both resazurin (Fig. 5A) and CV (Fig. 5B) assays. However, compounds 5 and 6 exhibited comparable concentration-dependent effects on the readouts of both assays across all cell types, although their effects were weaker than those of the reference compound 7 (torkinib).

**Fig. 6:**
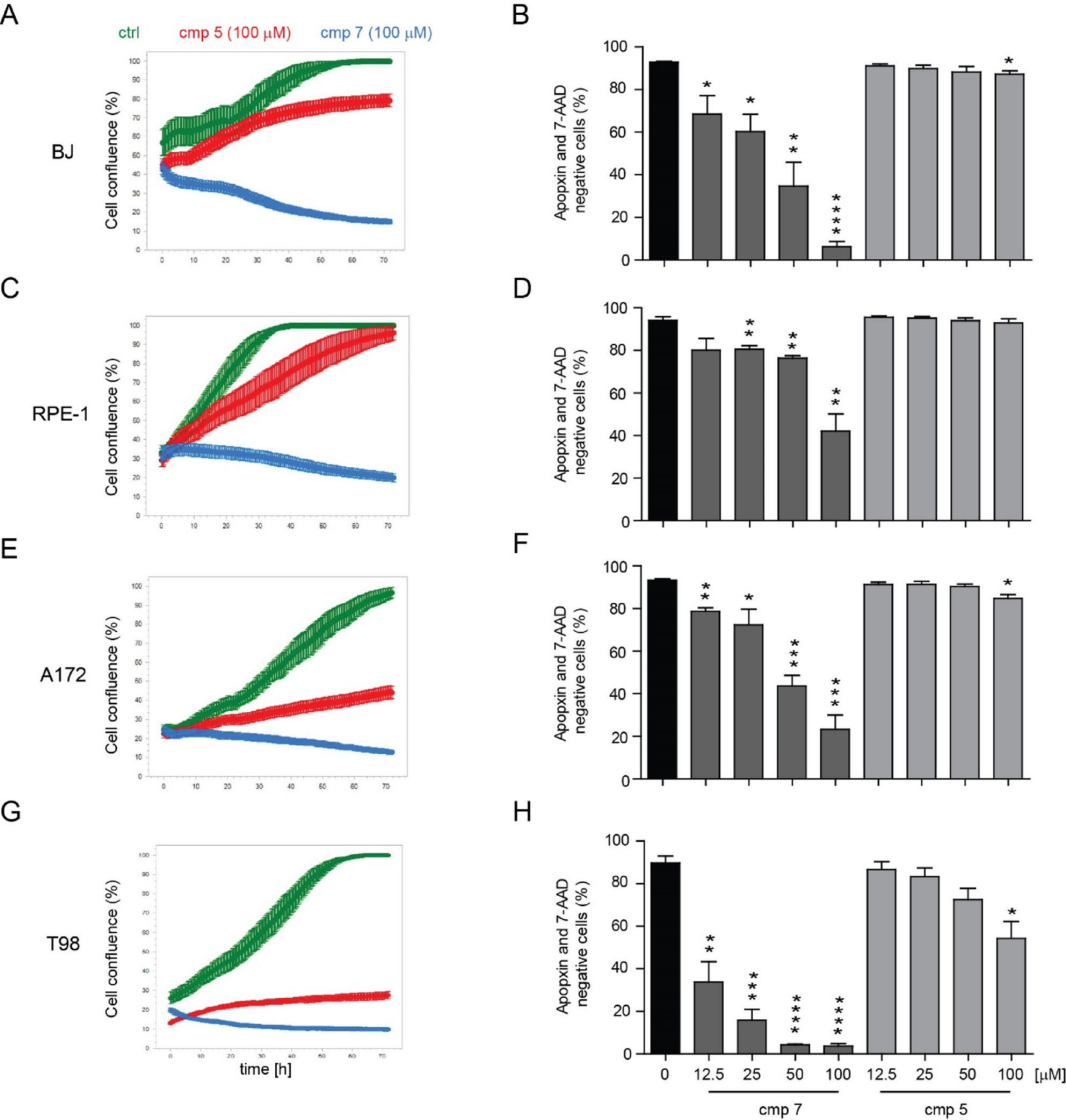
Cytostatic and cytotoxic effects of compounds 5 and 7. The antiproliferative effects of compounds 5 and 7 (100 *µ*M) on cell proliferation were detected in proliferating BJ (**A**), RPE-1 (**C**), and glioma A172 (**E**), and T98 (**G**) cells by time-lapse microscopy (0–72 h) using the Incucyte SX1 platform. The graphs show changes in cell confluence. Cell death in BJ (**B**), RPE-1 (**D**), A172 (**F**), and T98 (**H**) cell exposed to 0, 12.5, 25, 50, and 100 *µ*M concentration of compound 5 and 7 was determined by fluorescence-activated cell sorting analysis using apoptosis and necrosis markers Apopxin and 7-AAD, respectively. The graphs plot Apopxin- and 7-AAD-negative (live) population. The experiment was done in triplicate. Data were normalized to untreated cells and plotted as mean ± SEM. Statistical significance was determined using a two-tailed Student’s *t*-test. p-values ^****^p < 0.0001; ^***^p < 0.001; ^**^p < 0.01; *p < 0.05

To discriminate whether the antiproliferative effects of compounds 5 and 7 (torkinib) are cytostatic or cytotoxic, we employed time-lapse microscopy using the Incucyte SX1 platform to monitor cell proliferation (Fig. 6A, C, E, G) and cell death by fluorescently labeled apoptotic marker annexin V (Supplementary Figure S2A-I). Non-transformed BJ, RPE-1, and cancerous glioma A172, T98, U87, and U251 cells were exposed to compounds 5 and 7 at concentrations ranging from 10 to 100 *µ*M and monitored for 72 h (see Fig. 6 for cell growth curves plotted for 100 *µ*M concentration and Supplementary Figure S2A-I for end-point microscopic images). Although compound 5 suppressed the cell confluency for all tested cell types, non-transformed BJ and RPE-1 cells were less affected compared to A172 and T98 glioma cell lines (Fig. 6A, C, E, G). In contrast, compound 7 reduced cell confluency more significantly than compound 5 across all cell types tested, with the most pronounced effect in glioblastoma T98 cells (Fig. 6A, C, E, G). This difference compared to compound 5 was due to the significantly higher cytotoxicity of compound 7, as evidenced by the higher levels of the apoptotic marker annexin V (Supplementary Figure S2I).

Furthermore, to quantify dead cells for determination of cytotoxicity, we used fluorescence-activated cell sorting analysis of apoptotic and necrotic markers Apopxin and 7-AAD, respectively. BJ, RPE-1, A172, and T98 cells were exposed to compounds 5 and 7 at concentrations ranging from 12.5 to 100 *µ*M, and cell death was determined after 72 h (Fig. 6B, D, F, H). Compound 5 induced cell death only in a negligible number of BJ and A172 cells at 100 *µ*M, whereas a significant reduction in the number of live T98 cells was observed. The cytotoxicity of compound 5 was completely absent in RPE-1 cells. In contrast, treatment with compound 7 resulted in significant cell death across all cell lines tested, with RPE-1 cells being the most resistant and T98 cells the most sensitive.

**Fig. 7:**
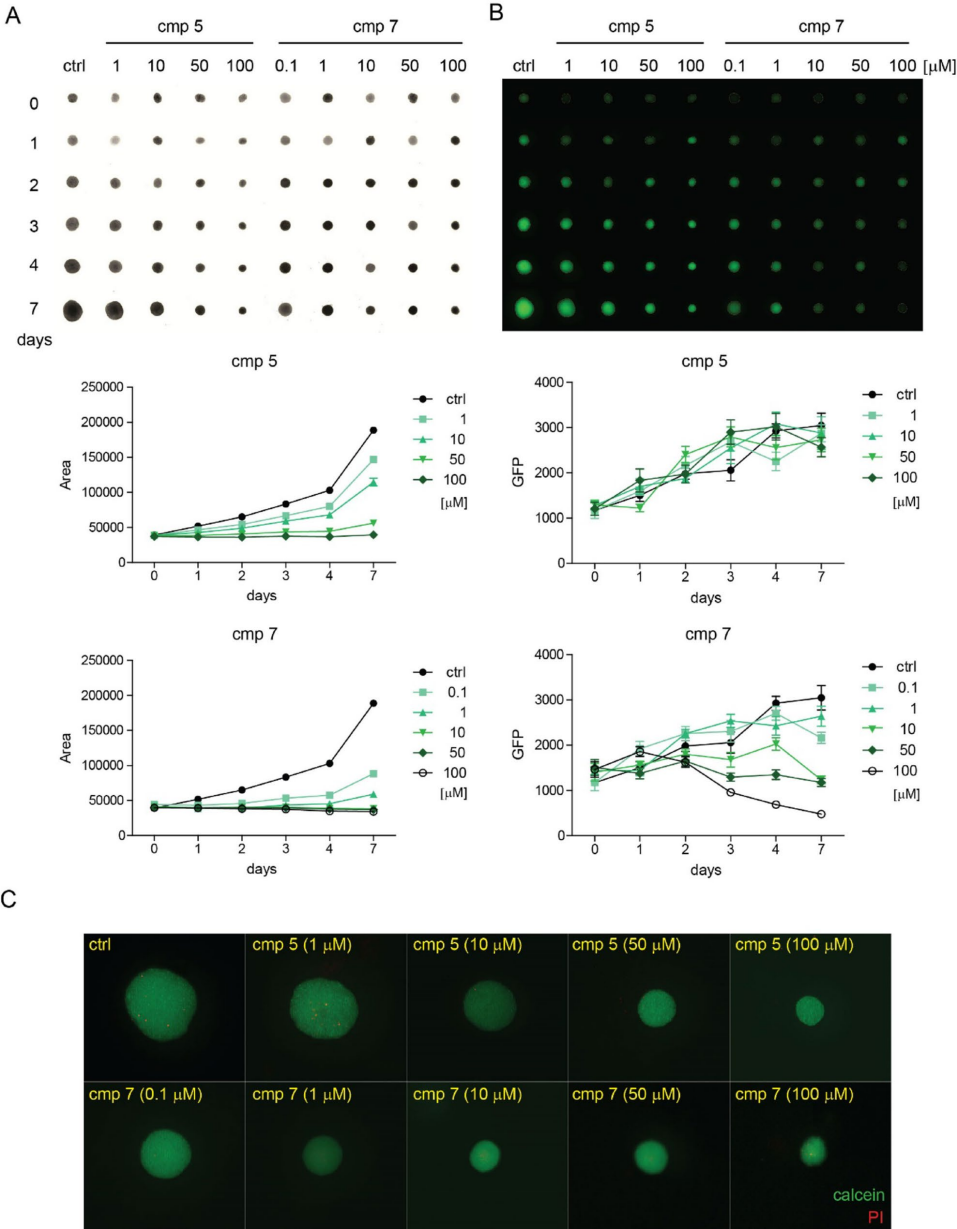
Compounds 5 and 7 affect cell growth in 3D spheroids. (**A**) Size, (**B**) green fluorescence intensity, and (**C**) calcein AM with propidium iodide (PI) staining of the 3D spheroids prepared from proliferating U87 cells expressing GFP were observed with ZEISS AxioZoom V.16 microscope. The representative 3D spheroids from 9 to 13 replicates per condition are shown; the size of individual rectangles is 1000 × 1000 *µ*m in A, B, and C. The graphs plot changes in the spheroid size and GFP fluorescent intensity (mean ± SEM; *n* = 9–13)

We further tested the ability of compounds 5 (1–100 *µ*M) and 7 (0.1–100 *µ*M) to suppress the growth of the glioma line U87 with ectopic expression of GFP in a 3D spheroid environment. A single dose of compound 5 (50 *µ*M) completely suppressed spheroid growth for at least 7 days. Compound 7 showed the same effect at a dose of 10 *µ*M (Fig. 7A). Ectopic GFP expression can serve as an indirect proxy for cell viability in spheroids. Treatment with compound 5 did not affect the intensity of the GFP signal, indicating cytostatic effect on spheroid growth. However, treatment with compound 7 decreased the GFP signal from a dose of 10 *µ*M (Fig. 7B), indicating rather cytotoxic effect. For direct detection of cell death, propidium iodide (PI), which stains dead cells, was combined with calcein AM, which stains live cells, at day 7. Again, compound 5 did not exhibit cytotoxicity, as demonstrated by positive calcein AM staining with the absence of PI staining. However, a similar effect was observed with compound 7, with the difference that the effect on spheroid growth was more pronounced at lower concentrations (1 and 10 *µ*M) than with compound 5 (Fig. 7C).

It can be concluded that both compounds had only a cytostatic effect on growth in a 3D environment over a wide concentration range.

In conclusion, compounds 1–4 did not affect cell growth in vitro. Compound 5 demonstrated concentration and cell line dependent cytostatic or cytotoxic activity, with higher effectiveness against cancerous glioma cells T98 than against cancerous glioma cells A172 and non-transformed BJ and RPE-1 cells. Compound 5 was cytotoxic only on glioma T98 cells. Compound 7 (torkinib) induced regulated cell death, in addition to suppressing proliferation, and demonstrated higher efficacy than compound 5, with similar effect on glioma cells A172, and T98, as well as non-transformed BJ and RPE-1 cells. Compound 5 showed a cytostatic effect in 2D and 3D cultures; however, compound 7 exhibited a low cytotoxic effect in 3D spheroids compared to 2D cultures of glioma cells. A similar higher drug resistance of 3D compared to 2D cultures was reported for paclitaxel and doxorubicin in dense 3D spheroids obtained from T-47D, BT-474, and BT-549 cells [24].

#### Compounds 5 and 7 affect the migration of glioma cells

**Fig. 8:**
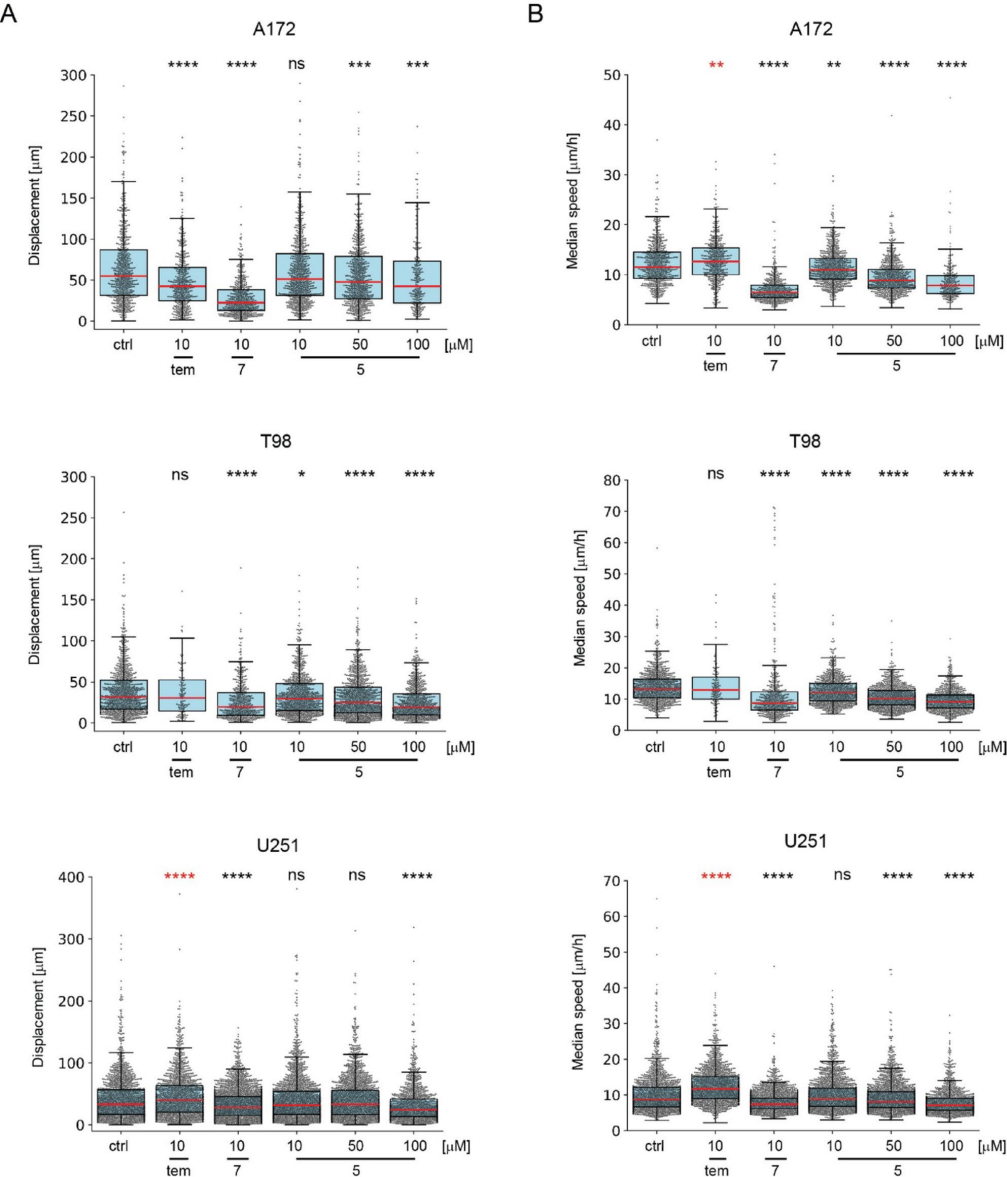
Effect of compounds 5 and 7 on migration of glioma cells. Glioma cells A172, T98, and U251 were exposed to compound 5 (10, 50, and 100 *µ*M), compound 7 (10 *µ*M), and temsirolimus (tem, 10 *µ*M) and followed by time-lapse microscopy (0–24 h) using the Incucyte SX1 platform. Two parameters were used to evaluate cell migration: (**A**) displacement and (**B**) median speed. Mann-Whitney U-test, ****, *p* < 0.0001; ***, *p* < 0.001; **, *p* < 0.01; *, *p* < 0.05. Black stars indicate conditions where displacement and median speed were significantly decreased compared to the control. Red stars indicate conditions where displacement and median speed were significantly increased compared to the control

During the time-lapse microscopy, cells exposed to compounds 5 and 7 showed slower migration compared to control cells. To quantify this observation, we analyzed random cell migratory activity using time-lapse microscopy, focusing on parameters such as cell displacement (Fig. 8A) and median speed (Fig. 8B, see Experimental Section for details). Glioma A172, T98, and U251 cells were exposed to compounds 5 (10, 50, and 100 *µ*M) and 7 (10 *µ*M), and cell migration was monitored using the Incucyte SX1 platform for 24 h. Rapalog temsirolimus (10 *µ*M) was used as a reference compound for mTOR inhibition via the FRB domain. Compound 7 reduced the speed and displacement of all glioma cell lines. In contrast, compound 5, at a concentration of 100 *µ*M, also significantly reduced the speed and displacement of glioma cells. Conversely, temsirolimus had no or an opposite effect on the median speed and displacement on glioma cells compared to compound 7 (torkinib), which is consistent with the study by Mecca et al. [25].

In summary, torkinib and its derivative compound 5 demonstrated a statistically significant inhibitory effect on the cell migration of three human glioma cell lines.

#### Compound 5 transiently induces autophagic flux

**Fig. 9:**
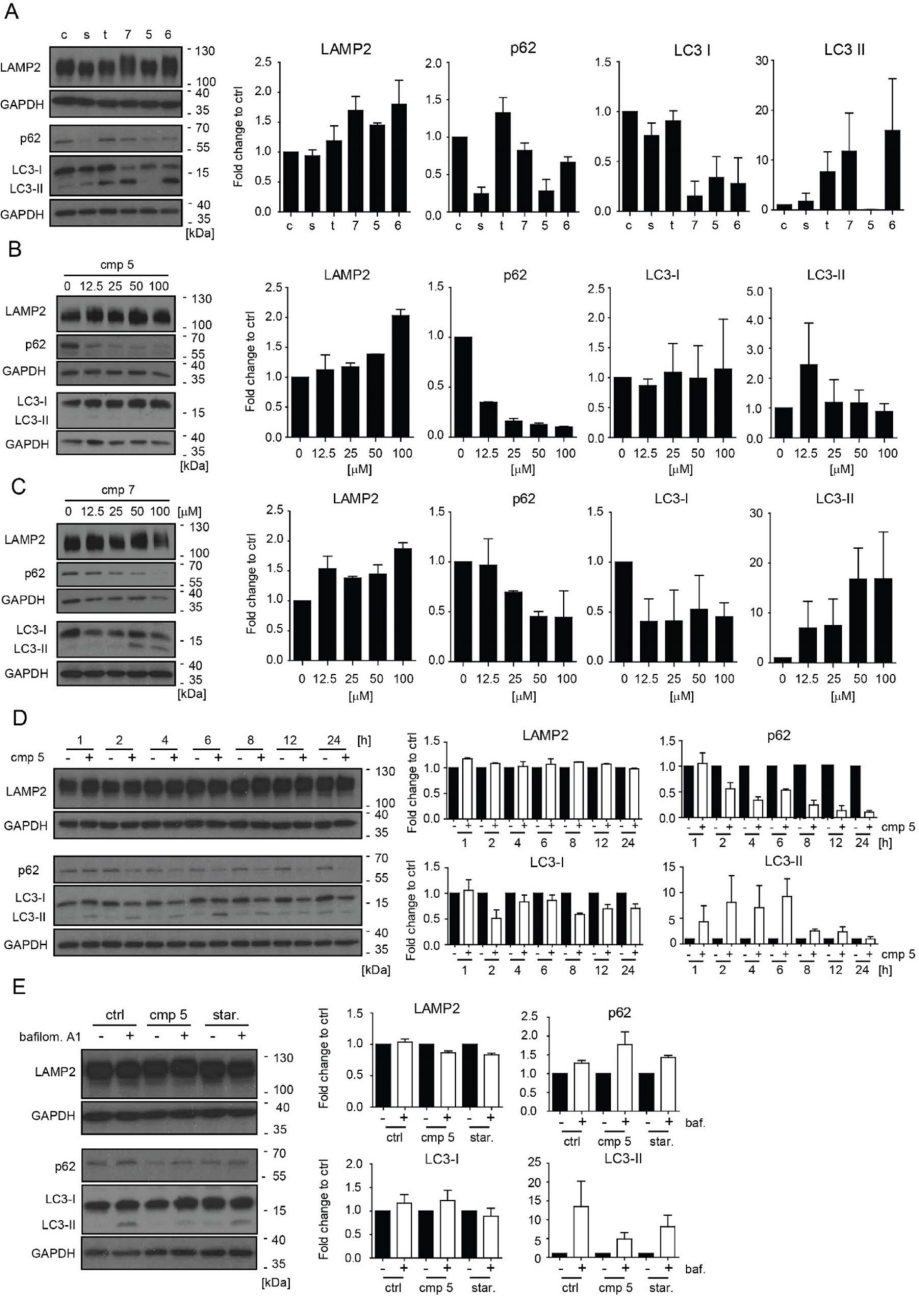
Effects of compounds 5 and 7 on autophagy in RPE-1 cells. (**A**) The effects of serum deprivation (starvation), temsirolimus (25 *µ*M), and compounds 5, 6, and 7 (100 *µ*M) were assessed in RPE-1 cells. RPE-1 cells were exposed to a 12.5, 25, 50, and 100 *µ*M concentration of compounds (**B**) 5 and (**C**) 7 for 24 h. (**D**) RPE-1 cells were harvested from 1 to 24 h after treatment with compound 5 (100 µM). (**E**) Bafilomycin A1 (100 nM) was added for 1 h after 23 h of cultivation in control cells, serum-deprived cells, and compound-**5**-treated cells (100 µM). For all experiments, the levels of LAMP2, p62, and LC3 were detected by immunoblotting using specific antibodies. GAPDH was used as a loading control. The ImageJ 1.48v program was used for quantitative analysis of immunoblots with GAPDH as a loading control. The experiment was done in duplicate

One of the biological effects of mTOR inhibitors is the induction of autophagy. First, we followed the impact of serum deprivation (starvation), temsirolimus (25 *µ*M), and compounds 5, 6, and 7 (100 *µ*M) on autophagy in autophagy-competent RPE-1 cells after 24 h (Fig. 9A). Autophagic activity was evaluated by monitoring LC3 processing, p62/SQSTM1 (p62) turnover, and LAMP2 protein abundance and glycosylation using immunoblotting and indirect immunofluorescence with specific antibodies.

Serum deprivation and exposure to temsirolimus, and especially to compounds 6 and 7, induced accumulation of LC3-II, the lipidated, membrane-associated form of LC3, indicating increased autophagosome production. This finding was supported by increased number of LC3 foci monitored by indirect immunofluorescence with LC3 antibodies (Supplementary Fig. 3A). In contrast, compound 5 did not induce a detectable LC3-II signal at 24 h (Fig. 9A). LC3-I levels were reduced upon treatment with compounds 5, 6, and 7, indicating that cytosolic LC3 is being efficiently lipidated. In contrast, LC3-I levels were not reduced after serum starvation and exposure to temsirolimus. Furthermore, to assess autophagy activity, we monitored the protein levels of p62, which acts as a cargo adaptor that links ubiquitinated proteins to the autophagy machinery. The decrease in p62, accompanied by a simultaneous increase in LC3-II upon serum starvation and upon exposure to temsirolimus and compounds 6 and 7, suggests activation of autophagy by these treatments. On the other hand, treatment with compound 5 was associated with a significant reduction in p62 protein levels after 24 h, indicating enhanced autophagic degradation. Although higher levels and glycosylation status of LAMP2 were detected in compounds 6 and 7 (Fig. 9A), changes in LAMP2 levels and glycosylation after treatment with temsirolimus, compound 5, or serum starvation were observed to be negligible compared to the control.

To assess whether the observed differences in autophagy activity between compounds 5 and 7 are concentration-dependent, we compared the levels of autophagic markers induced by compounds 5 and 7 across a concentration range of 12.5–100 *µ*M after 24 h of exposure (Fig. 9B, C). LC3-II level increased with increasing concentration of compound 7, indicating increased autophagosome production. In contrast, treatment with compound 5 led to a slight increase in LC3-II levels at a 12.5 *µ*M the concentration. LC3-I was reduced after treatment with compound 7, but not with compound 5. The level of p62 was reduced after administration of compound 5 at all tested concentrations, compared with compound 7, which reduced p62 only at 50 *µ*M indicating enhanced autophagic degradation. LAMP2 levels were similarly slightly increased by both compounds. All these findings suggest that the difference in the effect on autophagy activity is not due solely to the lower potency of compound 5.

Because in these experiments we compared the effects of compounds on autophagic markers at only one time point, we next monitored the dynamics of changes in autophagic markers at 1, 2, 4, 6, 8, 12, and 24 h after compound 5 treatment (100 µM). LC3-II levels increased rapidly within 1–6 h, then declined, returning to near-baseline levels by 24 h (Fig. 9D). A similar course was observed for the number of LC3 foci detected by indirect immunofluorescence (Supplementary Fig. 3B). LC3-I levels were only mildly reduced throughout the time course. In contrast, p62 protein levels progressively decreased over time, whereas LAMP2 abundance remained comparable to or slightly higher than in control cells (Fig. 9D). The decrease in p62 protein level was not caused by diminished p62 transcription, as p62 mRNA levels after 24 h of exposure to compound 5 were rather increased compared to control (Supplementary Fig. 3D). A transient increase of LC3 foci, which colocalized with p62 foci, was detected within 1–6 h of compound 5 treatment, followed by a decrease to control levels. No detectable changes of LAMP2 foci were detected (Supplementary Fig. 3B). These data indicate that autophagy was effectively activated by compound 5, with a strong but transient autophagic flux that rapidly returned to the normal state.

Furthermore, to determine the effect of compound 5 (100 µM) on autophagic flux, we next examined the effect of bafilomycin A1, an inhibitor of autophagosome–lysosome fusion, in control and serum-deprived cells (starvation), and compound **5**-treated cells. Bafilomycin A1 (100 nM) was added for 1 h after 23 h of normal cultivation, cultivation with compound 5 and cultivation by serum starvation. Under all three conditions, bafilomycin A1 led to a relative accumulation of both LC3-II and p62, consistent with an active autophagic flux (Fig. 9E), thus supporting the previous notion that autophagic flux is normalized after 24-hour treatment with compound 5. Increased number LC3 and p62 foci after bafilomycin A1 was also detected by indirect immunofluorescence, mainly in control and serum-deprived cells. No evident effects on LAMP2 foci were detected (Supplementary Fig. 3C).

Altogether, our findings indicate that compound 5 primarily promotes transient increase in autophagic flux rather than autophagosome accumulation characteristic for compounds 6 and 7.

#### Differential inhibition of AKT kinase activity with torkinib and compound 5

**Fig. 10:**
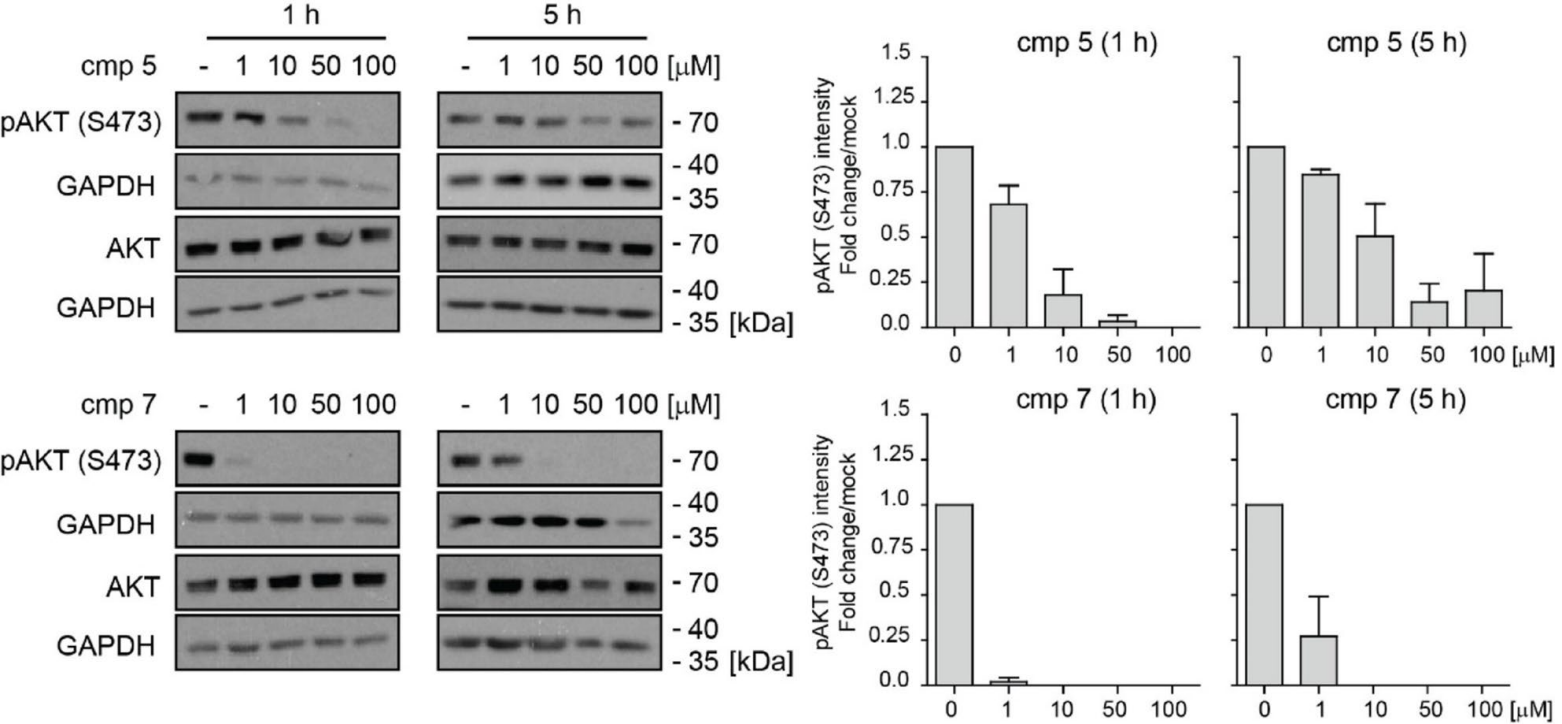
AKT inhibition by compounds 5 and 7. BJ cells were treated with compounds 5 and 7 in a concentration range of 1–100 *µ*M for 1 and 5 h. The level of AKT kinase Ser473 phosphorylation was detected using immunoblotting with specific antibodies. GAPDH was used as a loading control. Quantification of the pAKT (Ser473) signal expressed as a fold-change of the control (mean ± SEM; *n* = 2)

Since compound 5 suppressed the autophagy, we aimed to determine whether compound 5 directly inhibits AKT kinase. For this purpose, we compared compounds 5 and 7 across a concentration range of 1–100 *µ*M to assess their ability to inhibit the activation phosphorylation of AKT kinase on Ser473 in BJ cells exposed to either compound for 1–5 h. Compound 5 was by one order of magnitude less effective in AKT inhibition compared to compound 7 (Fig. 10). Thus, the autophagy inhibitory effect of compound 5 cannot account for the increased inhibition of the AKT kinase, suggesting a different mechanism responsible for this effect. In conclusion, compound 5 exhibited weaker inhibitory effect on AKT phosphorylation compared to compound 7 (torkinib).

#### Compound 5 exhibits a senomorphic activity including suppression of IL-1α, IL-6 and IL-8 expression

**Fig. 11:**
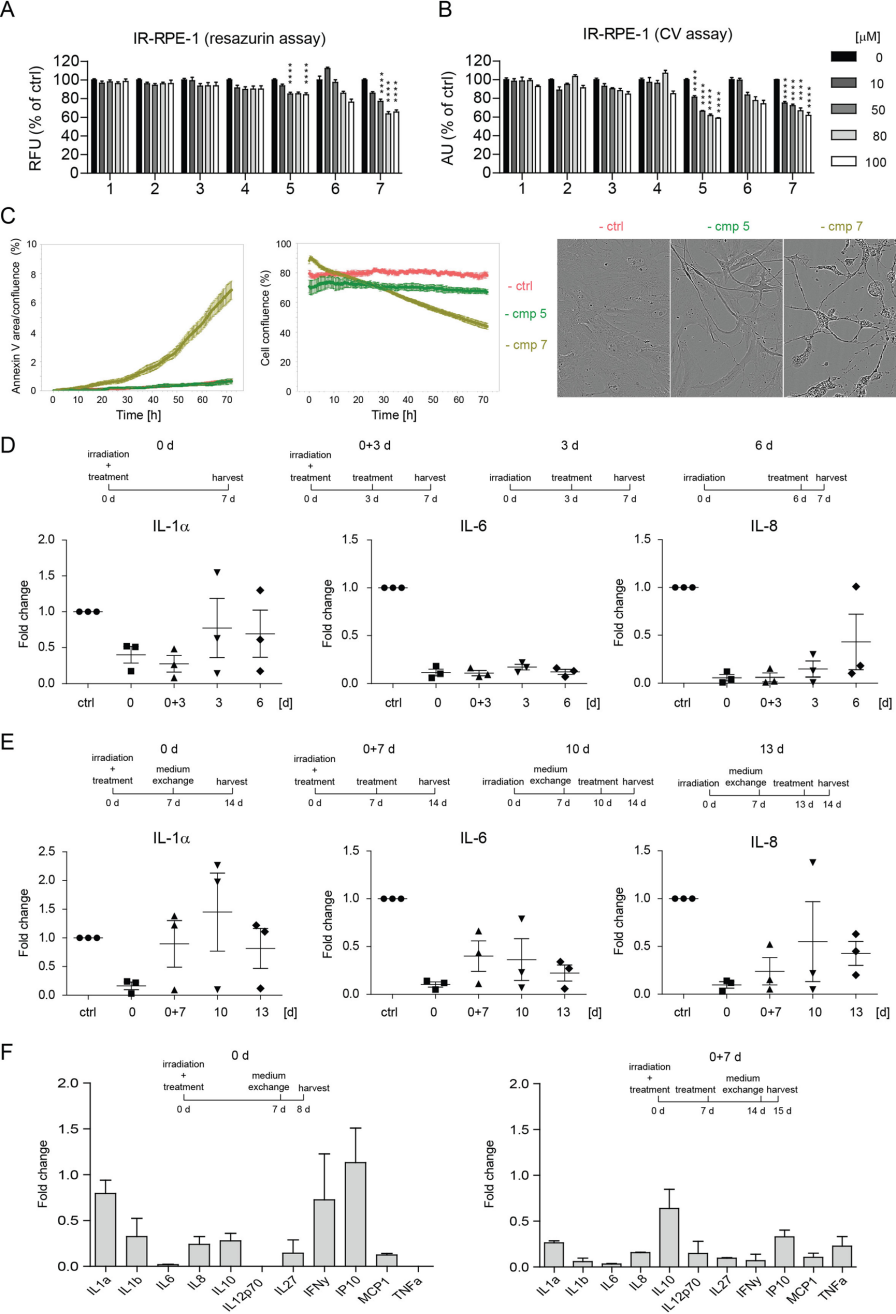
Effect of compounds 1–7 on viability and secretory activity of irradiation-induced senescent cells. Viability of irradiation-induced senescent RPE-1 cells (IR-RPE-1) exposed to compounds 1–7 at concentrations ranging from 10 to 100 *µ*M for 24 h was determined using the (**A**) resazurin and (**B**) crystal violet assays. (**C**) Cell death detected by fluorescently labeled annexin V staining of senescent IR-RPE-1 exposed to compounds 5 and 7 (100 *µ*M) was demonstrated by time-lapse microscopy (0–72 h) using the Incucyte SX1 platform. The graphs plot changes in cell confluence and cell death. Average cell confluence and annexin V staining positive area normalized to cell confluence with standard error from four images are shown. A representative 310 × 450 *µ*m region of end-point images (72 h) is presented. (**D**) Effects of compound 5 on the IL-1α, IL-6, and IL-8 transcript level quantified by real-time PCR in senescent cells (IR-RPE-1) harvested 7 days after irradiation with a schematic representation of the regimen of adding compound 5 to senescent cells. IR-RPE-1 cells were treated immediately after irradiation (0 d), and 3 days after irradiation (0 + 3 d), 3 days after irradiation (3 d), 6 days after irradiation (6 d) and harvested 7 days after irradiation. (**E**) Effects of compound 5 on the IL-1α, IL-6, and IL-8 transcript level quantified by real-time PCR in senescent IR-RPE-1 cells 14 days after irradiation with schematic representation of the regimen of adding compound 5 to senescent cells. IR-RPE-1 cells were treated with compound 5 immediately after irradiation (0 d), immediately and 7 days after irradiation (0 + 7 d), 10 days after irradiation (10 d), 13 days after irradiation (13 d), and harvested 14 days after irradiation (mean ± SEM; *n* = 3). (**F**) Detection of INFγ, IL-1α, IL-1β, IL-6, IL-8, IL-10, IL-12p70, IL-27, IP10, MCP-1, and TNFα protein levels by 11-Plex Panel in culture media conditioned by senescent IR-RPE-1 cells treated immediately and 7 days after irradiation. Culture media were harvested at day 8 and 15, respectively (mean ± SEM; *n* = 2)

Elimination of senescent cells or suppression of their adverse effects are emerging strategies of anticancer and anti-aging approaches [26]. Therefore, we first investigated whether compounds 1–7 would affect the viability of senescent cells. RPE-1 cells were induced to cellular senescence by ionizing radiation (IR-RPE-1) [27]. Senescent cell viability was assessed using resazurin and crystal violet assays after exposure to concentrations of compounds 1–7 ranging from 10 to 100 *µ*M for 24 h (Fig. 11A, B). Compounds 1–4 did not show any effect on the viability of the senescent cells in both assays. In resazurin assay based on determining metabolic capacity, compound 5 exhibited negligible effect, whereas compound 6 and 7 scored significantly from the concentration of 100 and 80 *µ*M, respectively. However, compounds 5 and 7 significantly decreased the readout of the CV assay, which could be due to cytotoxic (senolytic) or cell size (senomorphic) effects.

To distinguish between these two options, we monitored senescent IR-RPE-1 and DTX-BJ cells exposed to compounds 5 and 7 at concentrations ranging from 10 to 100 *µ*M with cell death marker, annexin V, using time-lapse microscopy for 72 h (Fig. 11C, Supplementary Figure S4A-E). Compound 5 demonstrated negligible annexin V positivity in the entire tested concentration range associated with a decrease in cell size at higher doses, indicating, in agreement with the resazurin assay, the absence of senolytic activity. In contrast, annexin V positivity increased significantly in senescent IR-RPE-1 cells treated with compound 7 at concentrations of 80 and 100 *µ*M, indicating a senolytic effect. Note also the decreasing intensity of senescence-associated beta-galactosidase staining, indicating decreased lysosomal compartment in senescent cells treated by compound 5 (Supplementary Figure S4F).

In the next step, to further evaluate the senomorphic effect of compound 5, we monitored the transcript levels of proinflammatory cytokines IL-1α, IL-6, and IL-8 using real-time PCR quantification. We compared several regimens of adding compound 5 to cells immediately after irradiation and during the development of IR-induced senescence for 7 and 14 days (see scheme in Fig. 11D, E). The most pronounced suppressive effect on transcript levels of IL-1α, IL-6, and IL-8 was observed when compound 5 was added immediately and 3 days after irradiation. Later addition of compound 5 resulted in significant downregulation of IL-6 only.

We further investigated the effect of compound 5 added immediately or 7 days after irradiation on the protein levels of proinflammatory cytokines in IR-RPE-1 cells using an 11-Plex Panel of cytokines, including INFγ, IL-1α, IL-1β, IL-6, IL-8, IL-10, IL-12p70, IL-27, IP10, MCP-1, and TNFα. The culture medium was exchanged with fresh medium without serum 24 h prior to harvest at day 8 or 15 (see scheme in Fig. 11F). Both early and late administration of compound 5 had a predominantly suppressive effect on most of the cytokines tested, except for INFγ and IP10 in IR-RPE-1 cells exposed to compound 5 immediately after irradiation (Fig. 11F), suggesting that compound 5 may affect the SASP once it has developed.

In conclusion, compounds 1–4 exhibited minimal or no effect on radiation-induced senescence. In contrast to a mild and nonspecific effect of compound 6 on senescent cells, compounds 5 and 7 showed a substantial effect on senescent cells, albeit in a different manner – compound 7 induced cell death, indicating senolysis. In contrast, compound 5 caused a decrease in cell size of radiation-induced senescent cells without inducing cell death, indicating a senomorphic effect. The suppression of pro-inflammatory components of SASP by compound 5 further supported this. Notably, the latter effect was observed not only immediately after irradiation but also in the course of senescence development.

### In vivo safety study

Acute toxicity determination of any compound is considered one of the major preconditions for successful drug development [28–30]. The promising approach for such purpose (i.e., LD_50_ determination) in mice is the application of a single dose wherein lethality can be first determined within 24 h of studies [31]. The toxicity of different compounds depends on their exposure routes, absorption, distribution, metabolism, and reaction with the targeted tissues within the animal’s body [32]. Intraperitoneal (*i.p.*) application constitutes a fast and easy way for the drug to enter the bloodstream, thereby inducing the effect. Structural differences, the results of physical-chemical analysis, and finally, in vitro outcomes highlighted compounds 5 and 7 as compounds worthy of in vivo investigation.

The results presented in Tables 4 and 5 demonstrate that the calculated LD_50_ value of compound 7 was the highest in female mice (939.51 mg/kg). A slightly lower LD_50_ value was recorded in female mice treated with compound 5 (686.86 mg/kg). Additionally, from the same Tables, it is evident that studied compounds 5 and 7 were less toxic for females than for males (686.86 mg/kg vs. 612.37 mg/kg; 939.51 mg/kg vs. 911.83 mg/kg, respectively), indicating sex differences.

**Table 4:** Effects of compound 5 on 24 h-survival in mice

Dose (mg/kg i.*p*.)	Total number dead/treated		LD_50_ (95% confidence limit) (mg/kg)	
	Male	Female	Male	Female
250.0	0/8	0/8	612.37 (520.72–720.15)	686.86 (591.76–797.23)
500.0	2/8	1/8		
750.0	6/8	5/8		
1000.0	8/8	8/8		

LD_50_ was calculated according to Litchfield & Wilcoxon

**Table 5:** Effects of compound 7 on 24 h-survival in mice

Dose (mg/kg i.*p*.)	Total number dead/treated		LD_50_ (95% confidence limit) (mg/kg)	
	Male	Female	Male	Female
500.0	0/8	0/8	911.83 (793.75–1047.48)	939.51 (844.33–1045.41)
750.0	2/8	1/8		
1000.0	5/8	5/8		
1250.0	8/8	8/8		

LD_50_ was calculated according to Litchfield & Wilcoxon

## Conclusion

Cellular senescence is the persistent cell cycle arrest in response to a variety of cellular stresses or DNA damage along with a proinflammatory response, mitochondrial dysfunction, and oxidative stress. Senescence plays a physiological role in embryonic development and wound healing. However, there is also the “dark side” of cellular senescence, as it is associated with various pathological conditions such as fatty liver disease, type 2 diabetes mellitus, tumorigenesis, and neurodegeneration. Fortunately, cellular senescence appears to be an adjustable process. Several signalling pathways are involved, e.g., p53, p38 MAPK, NF-κB or mTOR [33]. This study focuses on mTOR pathway.

The best-known mTOR inhibitor is rapamycin. In addition to its well-documented lifespan-prolonging effects in various organisms, rapamycin is involved in several clinical trials for the treatment of age-related diseases such as Alzheimer´s disease. However, rapamycin is not an ideal drug due to its physicochemical properties and side effects [34]. In our study, we aimed to discover novel small-molecule competitive mTOR kinase inhibitors with suitable physicochemical properties using two different in silico methods: virtual high-throughput screening of the ZINC database and fragment-based design. From the set of hit compounds, six lead compounds (1–6) were selected based on their commercial availability or synthetic feasibility. Torkinib (7) was also synthesized and used as a reference compound. Cell-based and biochemical evaluations revealed that torkinib-derivatived compounds 5 and 6 inhibit the phosphorylation of mTORC1 substrate p70 S6K. In contrast to rapamycin derivative temsirolimus, torkinib and its derivative compound 5 inhibited the migration of several glioma cell lines. Although the inhibitory potential of the novel compounds did not match that of torkinib, it remains debatable whether the most potent mTOR inhibitors necessarily have the most beneficial therapeutic effects. Importantly, the distinct biological profile of compound 5 may be related to its rationally optimized physicochemical properties. Compared to torkinib, compound 5 exhibits a more favorable CNS MPO score, lower lipophilicity, optimized molecular weight, and a more balanced polarity profile, reflecting a design strategy that considered drug-like properties in addition to target binding. These characteristics may contribute to its altered intracellular behavior and more moderate kinase inhibition profile, which is reflected biologically by its predominantly cytostatic rather than cytotoxic effects, weaker AKT inhibition, and transient, flux-competent autophagy induction. In contrast to the stronger and more sustained pathway suppression observed with torkinib, the biological activity profile of compound 5 appears to be associated with a more regulated modulation of mTOR signaling. This may underlie its distinct functional outcomes, including senomorphic activity and suppression of SASP components rather than direct senolytic effects. Together, these observations suggest that rational physicochemical optimization, as exemplified by compound 5, can influence biological activity profiles independently of maximal enzymatic potency, supporting the concept that drug-like properties may be as important as target affinity in the development of therapeutically relevant mTOR modulators. Finally, compound 5 suppressed senescence-associated expression of pro-inflammatory cytokines, including IL-6, both when administered simultaneously with cell irradiation and during radiation-induced senescence, which may be another pharmacologically practical benefit of this compound.

Although the in vivo analysis of torkinib and its derivative compound 5, demonstrated that compound 5 is slightly more toxic than torkinib, these findings indicate that compound 5 is still an interesting molecule worthy of further optimization.

## Experimental section

### Computational study

The commercially available ZINC database was used for virtual screening. Both screening and fragment-based design utilized previously validated docking procedures to assess the inhibition potential of compounds [17]. Complex of mTOR kinase with ligand PI-103 was downloaded from Protein Data Bank (PDB code: 4JT6). Prior to docking, magnesium ions were introduced. The protein underwent preparation using the Protein Preparation Wizard, involving the addition of hydrogen atoms and ionization at a pH of 7.4 ± 0.2. The binding site was defined as a box with an edge length of 10 Å and the center determined by co-crystalized compound PI-103. Docking was performed with Glide using a standard protocol, and ten poses were obtained for each ligand. Compounds were sorted according to the docking score values. The results were analyzed using PyMOL and Maestro, while the prediction of physicochemical properties was performed utilizing QikProp. Compounds selected for biological assay were purchased from chemical vendors or synthesized.

### Chemistry

All solvents, reagents, and the final compounds 1, 2, 3, and 4 were sourced commercially (Sigma Aldrich, Prague, Czech Republic; Activate Scientific, Prien, Germany; Penta Chemicals, Prague, Czech Republic; VWR, Stribrna Skalice, Czech Republic; MolPort SIA, Riga, Latvia) and they were used without any further purification.

Reactions were monitored using thin-layer chromatography (TLC) on Merck aluminum sheets coated with silica gel 60 F254 (Darmstadt, Germany). Visualization of plates was achieved using UV light (at 254 nm and 366 nm) or suitable staining reagents. Preparative column chromatography utilized silica gel 60 (with particle sizes ranging from 70 to 230 mesh, corresponding to 63 to 200 *µ*m, and a pore size of 60 Å). Flash chromatography was conducted on a Reveleris^®^Prep purification system from BÜCHI Labortechnik AG, Flawil, Switzerland, employing FlashPure BÜCHI Silica 40 *µ*m irregular columns (ranging from 4 to 80 g) or FlashPure BÜCHI C18 Silica 40 *µ*m irregular columns (ranging from 12 to 24 g). Melting points were determined using a Büchi M-565 melting point apparatus (BÜCHI Labortechnik AG, Flawil, Switzerland) and were reported without correction.

Nuclear magnetic resonance (NMR) spectra were acquired using a Bruker Avance NEO 500 MHz (with frequencies of 499.87 MHz for ^1^H and 125.71 MHz for ^13^C). Chemical shifts δ are reported in parts per million (ppm) and referenced to the signal center of solvent peaks (DMSO-*d*_*6*_: δ 2.50 ppm and 39.52 ppm for ^1^H and ^13^C, respectively; Chloroform-*d*: δ 7.26 ppm and 77.16 ppm for ^1^H and ^13^C, respectively; CD_3_OD: δ 3.31 ppm and 49.0 ppm for ^1^H and ^13^C, respectively). Coupling constants are expressed in Hz.

High-resolution mass spectra (HRMS) were acquired using a coupled LC-MS system consisting of Dionex UltiMate 3000 analytical LC system (ThermoFisher Scientific, Bremen, Germany) and Q Exactive Plus hybrid quadrupole-orbitrap spectrometer (ThermoFisher Scientific, Bremen, Germany). Heated electrospray ionization (HESI) was utilized as an ion source, with the following settings: sheath gas flow rate 40, aux gas flow rate 10, sweep gas flow rate 2, spray voltage 3.2 kV, capillary temperature 350 °C, aux gas temperature 300 °C, S-lens RF level 50. Positive ions were monitored in the 100–1500 m/z range at a resolution of 140,000. Mass spectra were processed using Xcalibur 3.0.63 software (ThermoFisher Scientific, Bremen, Germany). The non-calibrated purity of the final compounds was assessed via UHPLC–MS analysis using an Agilent Infinity II 1290 system coupled with a 6470 Series Triple Quadrupole mass spectrometer (electrospray ionization) and UV-VIS spectrophotometer (254 nm) as detectors (Agilent Technologies). Chromatographic separation was achieved on a Zorbax RRHD Eclipse plus C18 column (2.1 mm × 50 mm, 1.8 *µ*m) (Agilent Technologies) with eluents consisting of (A) 0.1% formic acid in water and (B) 0.1% formic acid in acetonitrile.

### Synthetic procedures and compounds' characterization

#### 3-Iodo-1-isopropyl-1*H*-pyrazolo[3,4-*d*]pyrimidin-4-amine (8)

To the solution of 3-iodo-1*H*-pyrazolo[3,4-*d*]pyrimidin-4-amine (200 mg, 0.76 mmol) in dry DMF (1.6 mL; inert atmosphere), K_2_CO_3_ (211 mg, 1.53 mmol) was added, and the mixture was stirred for 10 min. Isopropyl bromide (0.1 mL, 0.99 mmol) was added subsequently, and the reaction was carried out in a microwave reactor at 80 °C for 2 h. After cooling to ambient temperature, the mixture was extracted with EtOAc and cold water (2×), dried over Na_2_SO_4_, and concentrated to dryness. The crude product was purified by silica gel flash column chromatography (heptane : EtOAc 50–95% gradient) to give 8 as a white solid.

Yield 147 mg, 63%. Purity: 99%. M.p. 197–199 °C. ^1^H NMR (500 MHz, CDCl_3_) δ 8.31 (s, 1H), 6.06 (bs, 2 H), 5.08 (sept, *J* = 6.7 Hz, 1H), 1.54 (d, *J* = 6.7 Hz, 6 H). ^13^C NMR (126 MHz, CDCl_3_) δ 157.53, 155.85, 153.13, 104.20, 85.52, 49.83, 22.19. MS (ESI) calcd for C_8_H_11_IN_5_ [M + H]^+^ 304.00, found 304.1.

#### General procedure for preparation of substituted 4-amino-1-isopropyl-1*H*-pyrazolo[3,4-*d*]pyrimidines (5 and 6)

3-Iodo-1-isopropyl-1*H*-pyrazolo[3,4-*d*]pyrimidin-4-amine 8 (0.3–0.4 mmol) was dissolved in 1,4-dioxane (0.2 M). [1,1’-Bis(diphenylphosphino)ferrocene]palladium(II) dichloride in complex with dichloromethane (0.05 eq.) and appropriate boronic acid (1.2 eq.) were added subsequently. Finally, a saturated solution of Na_2_CO_3_ (0.2 M) was added, and the reaction mixture was stirred at 100 °C for 3 h. After the reaction was completed, the mixture was filtered through celite (in MeOH), and the solvent was evaporated *in vacuo*.

#### 3-(4-Amino-1-isopropyl-1*H*-pyrazolo[3,4-*d*]pyrimidin-3-yl)benzamide (5)

3-Iodo-1-isopropyl-1*H*-pyrazolo[3,4-*d*]pyrimidin-4-amine (85 mg, 0.28 mmol) reacted with (3-carbamoylphenyl)boronic acid (55 mg, 0.33 mmol) to give compound 5 as a white solid by the above-described general procedure. The crude product was purified by flash column chromatography on silica gel (DCM : MeOH 0–5% gradient).

Yield 65 mg, 78%. Purity: 98%. M.p. 237–239 °C. ^1^H NMR (500 MHz, DMSO-*d*_*6*_) δ 8.25 (s, 1H), 8.17 (s, 1H), 8.09 (s, 1H), 7.96 (d, *J* = 7.8 Hz, 1H), 7.79 (d, *J* = 7.6 Hz, 1H), 7.61 (t, *J* = 7.7 Hz, 1H), 7.46 (s, 1H), 5.13–5.04 (m, 1H), 1.50 (d, *J* = 6.7 Hz, 6 H). ^13^C NMR (126 MHz, DMSO-*d*_*6*_): δ 167.59, 158.11, 155.50, 153.41, 142.79, 134.98, 133.16, 130.95, 129.03, 127.44, 127.41, 97.49, 48.19, 21.79. HRMS (ESI) calcd for C_15_H_17_N_6_O [M + H]^+^ 297.1458, found 297.1451.

#### 1-Isopropyl-3-(5-methoxy-1*H*-indol-2-yl)-1*H*-pyrazolo[3,4-*d*]pyrimidin-4-amine (6)

3-Iodo-1-isopropyl-1*H*-pyrazolo[3,4-*d*]pyrimidin-4-amine (140 mg, 0.46 mmol) reacted with (1-(*tert*-butoxycarbonyl)-5-methoxy-1*H*-indol-2-yl)boronic acid (270 mg, 0.92 mmol) to give compound 6 as a red solid by the above-described general procedure. The crude product was purified by flash column chromatography on silica gel (heptane : EtOAc 30–50% gradient).

Yield 100 mg, 67%. Purity: 98%. M.p. 77–79 °C. ^1^H NMR (500 MHz, CDCl_3_) δ 9.16 (s, 1H), 8.37 (s, 1H), 7.32 (d, *J* = 8.8 Hz, 1H), 7.11 (d, *J* = 2.4 Hz, 1H), 6.92 (dd, *J* = 8.8, 2.4 Hz, 1H), 6.81 (d, *J* = 1.5 Hz, 1H), 6.29 (bs, 2 H), 5.19 (sept, *J* = 6.7 Hz, 1H), 3.87 (s, 3 H), 1.58 (d, *J* = 6.7 Hz, 6 H). ^13^C NMR (126 MHz, CDCl_3_) δ 158.14, 155.66, 154.80, 153.66, 136.86, 131.82, 131.42, 129.35, 114.00, 112.18, 102.40, 101.99, 98.55, 55.93, 49.13, 22.14. HRMS (ESI) calcd for C_17_H_19_N_6_O [M + H]^+^ 323.1620, found 323.1638.

#### 2-(4-Amino-1-isopropyl-1*H*-pyrazolo[3,4-*d*]pyrimidin-3-yl)-1*H*-indol-5-ol (7)

Compound 6 (160 mg, 0.50 mmol) was dissolved in dry DCM (2.5 mL) and cooled to -10 °C. Boron tribromide (0.1 mL, 1.0 mmol) was added to the reaction, and the mixture was stirred at -10 °C for 1 h. Afterwards, the reaction was quenched with water and stirred for an additional 30 min. DCM was added, and the organic layer was separated. Then, the pH of the water layer was adjusted to 9 with saturated Na_2_CO_3_ and extracted with EtOAc. The organic layers were dried over anhydrous Na_2_SO_4_ and concentrated *in vacuo*. The crude product was purified by flash column chromatography on silica gel (DCM : MeOH 0–20% gradient) to give 7 as a white solid.

Yield 49 mg, 32%. Purity: 97%. M.p. 144–146 °C. ^1^H NMR (500 MHz, CD_3_OD) δ 8.23 (s, 1H), 7.30 (d, *J* = 8.7 Hz, 1H), 7.01 (d, *J* = 2.3 Hz, 1H), 6.77 (dd, *J* = 8.7, 2.3 Hz, 1H), 6.70 (s, 1H), 5.12 (sept., *J* = 6.7 Hz, 1H), 1.58 (d, *J* = 6.7 Hz, 6 H). ^13^C NMR (126 MHz, CD_3_OD) δ 159.86, 156.58, 154.12, 152.26, 139.14, 133.73, 132.10, 130.93, 114.19, 113.11, 105.56, 102.74, 99.36, 50.34, 22.23. HRMS (ESI) calcd for C_16_H_17_N_6_O [M + H]^+^ 309.1464, found 309.1482.

### Physicochemical analysis

#### Calculation of Mw, tPSA, HBDs and CNS MPO

The Mw, tPSA, and HBD values were calculated using ACDLAbs PhysChem Suite 14.0 software (Advanced Chemistry Development, Inc., Toronto, ON, Canada). The CNS MPO scores were calculated using the previously published method by Wager et al. [21, 35].

#### Partition coefficient logP and distribution coefficient logD (pH 7.4)

For the determination of logD, a 10 nM sodium phosphate buffer of pH 7.4 was used. Before logP or logD were determined, all used solvents were mutually saturated at the temperature of the experiment (21 °C). To do this, large stock bottles, one containing *n*-octanol and a sufficient quantity of water/buffer, and the other containing water/buffer and a sufficient quantity of *n*-octanol, were shaken for 30 min on a mechanical shaker and then left to stand long enough to allow the phases to separate. The constant quantity of the tested compound (approx. 0.50 mg) and 1:1 volume ratio of saturated solvents (250 *µ*L) were added to the test vessels. The test vessels were placed in a mechanical shaker (Multi Reax, Heidolph Instruments, Schwabach, Germany) and shaken for 30 min at the highest speed. Two phases were separated by centrifugation (16,873 × g for 5 min; Eppendorf 5418 Microcentrifuge, Prague, Czech Republic). The samples from both organic and aqueous phases were transferred to separate vials for LC-MS analysis. The exact concentration of tested compounds was determined by UHPLC-DAD-MS (UHPLC Infinity II 1290, detection by DAD and QqQ 6470; Agilent Technologies, CA, USA), separation on Arion C18 polar column (100 × 2.1 mm, 2.1 *µ*m; Chromservis, Czech Republic). The logP/logD values were calculated using the total quantity of compound present in both phases.

#### Dissociation constants

The dissociation constants were acquired by spectrophotometric titration, using spectrophotometer Agilent Cary-60 (Agilent Technologies, CA, USA), pH meter WTW InoLab_IDS Multi 9430 (WTW, Czech Republic) and pH electrode SenTix^®^ Mic (WTW, Czech Republic). Data was analyzed using the software GraphPadPrism 8.0 (GraphPad Software, CA, USA). 20 *µ*L of compound stock solution (2 mg/mL) and 20 L of 0.1 M hydrochloric acid were added into the cuvette with ultrapure water (total volume 3.5 mL). While stirred at 20 °C, the sample was titrated with 0.1 M sodium hydroxide. Absorption spectra (190–500 nm) were acquired at different pH values after each base addition. Absorbance at the chosen wavelength was plotted as a function of pH, and the pKa value was determined from the inflection point in the given plot.

#### Solubility

Nephelometry assays on the NEPHELOstar microplate instrument (BMG Labtech, Offenburg, Germany) were performed to determine compound solubility. Analyzed compounds were dissolved in ultrapure water or sodium phosphate buffer (pH 7.4) to obtain stock solutions in the concentration of 160 *µ*g/mL, and subsequently, each suspension was sonicated at full power with Hielscher UP100H needle ultrasonic processor (Teltow, Germany). Following sonication, the suspension was loaded into NEPHELOstar instrument injector A, while ultrapure water or sodium phosphate buffer was loaded into injector B. Compound dilution was performed in 48 wells in 0 to 160 *µ*g/mL range. Followed by 30-second shaking, each well was scanned with 80% laser power in matrix 3 × 3, beam width 2 mm. Obtained data were evaluated in GraphPad Prism 7.03 (GraphPad Software, San Diego, CA, USA) using segmental linear regression.

### In vitro evaluation

#### mTOR binding assay

All chemicals (i.e., FRAP1, kinase tracer 314, LanthaScreen^®^ Eu-anti-GST Tag antibody, and mTOR kinase binding buffer) were purchased from ThermoFisher Scientific, USA. To evaluate the binding of newly synthesized compounds to mTOR, LanthaScreen^®^ Eu Kinase Binding TR-FRET Assay was used. All compounds were diluted in mTOR binding buffer and preserved at 4 °C until analyzed. The reaction mixture consisted of 5 *µ*L of a compound, 5 *µ*L of a kinase/antibody mixture (mTOR/Eu-labelled anti-Tag antibody; 2 nM/2 nM), and 5 *µ*L of a tracer (Alexa Fluor^®^ 647-labeled; 20 nM). The values in the brackets indicate the final concentrations of a compound in a well. The mixtures were incubated for 1 h at RT. Then, the fluorescence of the donor (EX/EM = 340/620 ± 10 nm) and the acceptor (EX/EM = 340/665 ± 8 nm) was detected, and the emission ratio of acceptor/donor fluorescence was calculated. Measurements were performed using an Infinite M200 well-plate reader (Tecan, Salzburg, Austria). The IC_50_ values were calculated using OriginPro 9 (OriginLab, USA).

#### Chemicals

The following chemicals were used: temsirolimus (Sigma, cat. no. PZ0020), propidium iodide (Thermo Fisher Scientific, cat. no. P21493), DMSO (Sigma, cat. no. D2650), DAPI (4′,6-diamidino-2-phenylindole) [Sigma-Aldrich/Merck (Darmstadt, Germany, cat. no. D9542)], Mowiol (Sigma-Aldrich/Merck, cat. no. 81381), bovine serum albumin (BSA) (Sigma, cat. no. A9647), resazurin (Sigma, cat. no. R7017), crystal violet (the British Drug Houses Ltd. Poole UK), calcein AM (Invitrogen, C3099) and AnnexinV-Dy647 (Apronex/Exbio, cat. no. RC-ANXF-T020).

#### Cell cultures

All cell lines used in experiments were obtained from the American Type Culture Collection (ATCC, Manassas, VA, USA). Human telomerase-immortalized retinal pigment epithelial cells hTERT RPE-1 (RPE-1), human glioblastoma A-172 (A172), T98G (T98), U-87 MG (U87), U-251 MG (U251), were cultured in Dulbecco’s modified Eagle’s medium (DMEM, Thermo Fisher Scientific, Waltham, MA, USA) containing high glucose (4.5 g/L). Primary human foreskin BJ fibroblasts (initial population doubling 28) were cultured in Dulbecco’s modified Eagle’s medium (DMEM, Thermo Fisher Scientific, Waltham, MA, USA) with low glucose concentration (1 g/L). Both culture media were supplemented with 10% fetal bovine serum (FBS, Gibco/Thermo Fisher Scientific), 100 units/mL of penicillin, and 100 *µ*g/mL of streptomycin (Gibco/Thermo Fisher Scientific). Cells were maintained at 37 °C in a 5% CO_2_ atmosphere with 95% humidity. All glioma cell lines, BJ and RPE-1 cells were authenticated by DNA fingerprint analysis (GENERI BIOTECH, Hradec Kralove, Czech Republic). All cell lines were free of mycoplasma (tested regularly, MycoAlert, Lonza).

U87-GFP cells were established via stable transfection of U-87 MG cells with the pEGFP-C1 vector (Addgene) using Lipofectamine 2000 (Thermo Fisher Scientific), following the manufacturer’s protocol. GFP-positive cells were subsequently isolated by FACS, and stable expression was maintained using selection with G418 (600 µg/mL; Sigma-Aldrich, St. Louis, MO, USA).

#### Antibodies

For immunoblotting, the following primary and secondary antibodies were used: rabbit polyclonal p70 S6 kinase (#9202), rabbit monoclonal phospho-p70 S6 kinase (Thr389) (#9234), rabbit monoclonal AKT kinase (#4691), rabbit monoclonal phospho-AKT kinase (Ser473) (#4060), rabbit monoclonal LC3B (#3868), mouse monoclonal p62/SQSTM1 (#88588) obtained from Cell Signaling Technology, Inc., Danvers, MA, USA; mouse monoclonal LAMP2 (555803) BD Pharmingen, Inc., San Diego, CA, USA; and mouse monoclonal anti-GAPDH (GTX3066) purchased from GeneTex, Inc., Irvine, CA, USA; goat anti-mouse (170–6516) or anti-rabbit IgG (H + L)-HRP conjugate (170–6515) purchased from BioRad, Hercules, CA, USA.

For indirect immunofluorescence, the following primary antibodies were used: rabbit monoclonal LC3B (#3868), mouse monoclonal p62/SQSTM1 (#88588) obtained from Cell Signaling Technology, Inc., Danvers MA, USA; mouse monoclonal LAMP2 (555803) BD Pharmingen, Inc., San Diego, CA, USA. The following secondary antibodies were used: goat antirabbit Alexa Fluor 488-conjugated (GAR488) and goat antimouse Alexa Fluor 568-conjugated (GAM568) (both Invitrogen/Thermo Fisher Scientific, Waltham, MA, USA, cat. no A-11034 and A-11031, respectively).

#### SDS-PAGE and immunoblotting

Cells were collected at post-treatment, washed with PBS, and lysed in SDS sample lysis buffer (SLB) consisting of 2% SDS, 63 mM Tris–HCl, pH 6.8, and 10% glycerol. Following sonication and centrifugation, the protein concentration was determined using a BCA assay (Pierce Biotechnology, Rockford, IL, USA). Samples were adjusted to an equal protein amount (40 *µ*g) by the addition of SLB containing dithiothreitol (1% final concentration) and bromophenol blue (0.02% final concentration) and subsequently separated by SDS–PAGE in Tris-glycine-SDS buffer (BioRad, 1610772). Proteins were then transferred onto a nitrocellulose membrane (0.45 *µ*m NC, Amersham™, GE Healthcare Life Sciences) via wet transfer in Tris-glycine buffer (BioRad, 1810704) supplemented with 10% methanol (Sigma, 59304). Following blocking with 5% non-fat milk in PBS/Tween-20, specific antibodies and horseradish peroxidase (HRP)-conjugated secondary antibodies were used for protein detection. Peroxidase activity was visualized using ECL detection reagents (Thermo Fisher Scientific, Waltham, MA, USA).

#### Cell proliferation assays

Cell proliferation was assessed using crystal violet [36] and resazurin assays [37] along with time-lapse microscopy [17]. Cells were seeded in 96-well plates at different densities depending on the cell type and condition: 2850 cells per well for senescent RPE-1 (IR-RPE-1), 3350 cells per well for proliferating RPE-1, and 5000 cells per well for glioblastoma A172, T98, U87, U251 cell lines, and proliferating and senescent BJ cells. The seeding of cells was performed 24 h before treatment.

#### Crystal violet assay

For the crystal violet assay, the cells underwent two washes with 150 *µ*L of PBS, followed by staining with 30 *µ*L of 0.5% (w/v) crystal violet in 20% methanol for 15 min. Subsequently, the plates were washed five times with ddH_2_O and allowed air dry overnight. To solubilize the crystal violet, 75 *µ*L of 0.2% Triton X-100 (Sigma) in PBS was added for 15 min. Absorbance was then measured at 595 nm using a microplate reader (Envision, PerkinElmer, Waltham, MA, USA). The absorbance of crystal violet in treated cells was normalized to the absorbance in untreated cells and expressed as a percentage. Each condition was assessed in triplicate or more.

#### Resazurin assay

For the resazurin assay, 100 *µ*L of resazurin solution (stock solution 30 mg/mL; Sigma, St. Louis, MO, USA), diluted tenfold in the culture medium, was added to each well, replacing 200 *µ*L of the culture medium. The cells were then incubated at 37 °C for 1 to 3 h. Fluorescence was measured using a microplate reader (PerkinElmer, Waltham, MA, USA). The fluorescence values obtained were normalized to those of untreated cells. Each condition was measured in triplicate or more.

#### Detection of regulated cell death by fluorescence-activated cell sorting

Both adherent and floating cells were harvested by 0.25% trypsin/EDTA and collected by centrifugation (500 rpm, 4 °C, 5 min). Cell pellets were resuspended in 200 *µ*L Assay buffer with Apopxin and 7-AAD (Apoptosis/necrosis detection kit, ab176749, Abcam). Cells were incubated in the dark at room temperature for 30 min and analyzed by the BD FACSVerseTM flow cytometer (BD Biosciences, Franklin Lakes, NJ). Data were analyzed using FlowJo 10 software (FlowJo LLC, Ashland, OR).

#### Determination of spheroid growth

96-well plates pre-coated with polyHEMA (5 mg/mL) were seeded with 1000 U87-GFP cells per well [38]. To form spheroids, well plates were centrifuged at 200×g at RT for 5 min and then incubated on a rotary shaker in the cell culture incubator at 37 °C, 5% CO_2_ for 3 days. Three days after the seeding, 100 *µ*L of the conditioned medium was exchanged with a fresh medium with two times concentrated compound 5 and 7 (50 mM stock solution).

The spheroid growth, size, and GFP signal intensity were observed daily by scanning bright-field and fluorescence for 5 days, and also at day 7 post-treatment using ZEISS AxioZoom V.16 microscope equipped with PlanNeoFluar Z 1.0× /0.25 objective and eGFP Filter set 38 HE (each spheroid in a hexaplicate). Images were analyzed using Fiji distribution of Image and made-to-measure macros.

At day 7, spheroids were stained with propidium iodide (PI) to detect dead cells and calcein AM for the detection of live cells. Firstly, a dye solution of 8 *µ*M calcein AM and 60 *µ*M PI in the cell culture medium without phenol red was prepared. Then, 100 *µ*L of cell culture medium was substituted in each well with 100 *µ*L of the dye solution to the final concentration of calcein AM 4 *µ*M and PI 30 *µ*M. After the incubation for 1 h in the cell culture incubator (37 °C, 5% CO_2_), 100 *µ*L of medium in the well was exchanged for 100 *µ*L of fresh medium without phenol red. Fluorescently labeled spheroids were captured by ZEISS AxioZoom V.16 microscope equipped with PlanNeoFluar Z 1.0× /0.25 objective, ApoTome.2 for optical sectioning and with applied eGFP Filter set 38 HE (for detection of calcein signal) and Cy3 Filter set 63 HE (for detection of PI signal). Images were processed in ImageJ software.

#### Time-lapse microscopy

To enable real-time observation of cellular dynamics, the Incucyte SX1 Live-Cell Analysis System (Sartorius, Göttingen, Germany) was employed. This system was housed within an incubator set at 37 °C, with a 5% CO_2_ atmosphere and 95% humidity. Images were captured at a resolution of 1408 × 1040 pixels with a pixel size of 1.24 microns, using a 10× objective lens in phase contrast, red fluorescence (excitation: 585 ± 20 nm, emission: 635 [625, 705] nm, acquisition time: 100 ms) channels, at 60-minute intervals over a period of 72 h. The initial image was acquired 30 min after the addition of compounds. Image analysis for cell confluence was conducted using Incucyte software (version 2022 A). Apoptotic cells were stained using Annexin V – Dy647 (Apronex, diluted 1:2000).

For cell movement analysis, cell nuclei were stained with 0.5 *µ*M SiR-DNA (Spirochrome, 251SC007), and images were acquired at 15-minute intervals over a period of 24 h. Images were pre-processed by the Lucas-Kanade algorithm implemented in the Image Stabilizer ImageJ plugin [39] and cropped to 1200 × 900 pixels to eliminate edge artifacts. Tracking was performed by the StarDist Versatile (fluorescent nuclei) model [40] in the TrackMate plugin [41] of ImageJ/Fiji [42] using LAP Tracker (linking 50 *µ*m, gap closing 50 *µ*m, 2 frames). Nuclei tracked for less than 2/3 of the experiment duration were not analyzed.

#### Detection of autophagy

For microscopic analysis of autophagy, RPE-1 cells were seeded onto 40-mm culture dishes with 10-mm coverslips (10,000 cells/cm^2^) and cultured overnight. RPE-1 cells were exposed to compound 5 and 7 (conc. 12.5–100 *µ*M), compound 6 (100 *µ*M), temsirolimus (25 *µ*M), or starved in medium without serum for 24 h. Coverslips with RPE-1 cells were fixed with 4% formaldehyde in PBS for 15 min, washed three times with PBS for 5 min, permeabilized in 0.2% Triton X-100 in PBS for 10 min, blocked in 10% FBS in PBS for 30 min, and incubated with primary (LC3B, p62, and LAMP2) antibodies for 1 h at RT. After that, cells were washed three times in PBS for 5 min each, secondary antibodies (GAR488 and GAM568) were applied in RT for 1 h, and slides were mounted with Mowiol containing DAPI. The wide-field images were acquired on the Leica DM6000 fluorescent microscope. To detect autophagic foci, the background in the channel was subtracted in ImageJ/Fiji [42].

#### Induction of cellular senescence

Proliferating RPE-1 cells were exposed to a single dose of X-rays (20 Gy) using Pantak HF160 (Gulmay, Surrey, UK) X-ray instrument equipped with Pantak Seifert HF320 generator, MXR-161 X-ray tube (Comet AG, Flamatt, Switzerland), and an aluminum filter using current 1–10 mA. After irradiation (IR-RPE-1), the cells were cultured for an additional 7 or 14 days until the development of the senescent phenotype [43, 44]. Proliferating BJ cells were exposed to a single dose of 5 nM docetaxel (DTX-BJ) and were cultured for an additional 7 days until the development of the senescent phenotype [44].

#### Senescence-associated beta-galactosidase activity

Senescence-associated beta-galactosidase activity was performed according to Dimri et al. [45]. Briefly, proliferating cells and senescent cells were fixed with 0.5% glutaraldehyde at room temperature for 15 min. Then, cells were washed twice with 1 mM MgCl_2_/PBS and incubated with X-Gal staining solution at 37 °C for 4 h. The staining was terminated by three consecutive washes with ddH_2_O. Finally, the cells were kept dry for 15 min and mounted with Mowiol containing DAPI. For imaging, the Leica DM6000 fluorescent microscope was used, equipped with a HC PLAN APO 20×/0.70 DRY PH2 objective and a color CCD camera (Leica DFC490; Leica Microsystems GmbH, Wetzlar, Germany).

#### Real-time quantitative reverse transcription PCR

Senescent cells were harvested using RLT lysis buffer (RNeasy Mini Kit Qiagen, Germantown, MD, USA) with DTT (20 *µ*L of 2 M DTT/1 mL RLT), and then total RNA was isolated according to the manufacturer’s protocol. The amount of RNA was measured by NanoDrop 2000 (Thermo Fisher Scientific, Carlsbad, CA, USA). Total RNA was reverse-transcribed into cDNA with random hexamer primers and MultiScribe reverse transcriptase (Applied Biosystems, Foster City, CA, USA). Expression levels of IL-1α (forward 5´-TGTATGTGACTGCCCAAGATGAAG-3´ and reverse 5´-AGAGGAGGTTGGTCTCACTACC-3´), IL-6 (forward 5´-AGACAGCCACTCACCTCT-3´ and reverse 5´-TTCTGCCAGTGCCTCTTT-3´), IL-8 (forward 5´-TTGGCAGCCTTCCTGATTTC-3´ and reverse 5´-TCTTTAGCACTCCTTGGCAAAAC-3´), and p62 (forward 5´-ATCGGAGGATCCGAGTGT-3´ and reverse 5´-TGGCTGTGAGCTGCTCTT-3´) were determined by qPCR relative quantification (Applied Biosystems SYBRTM Select Master Mix, Applied Biosystems 7300 Real-Time PCR System with SDS software, Thermo Fisher Scientific). The relative quantity of cDNA was determined by the ΔΔCt method [46]. Data were normalized to GAPDH (forward 5´-GTCGGAGTCAACGGATTTGG-3´ and reverse 5´-AAAAGCAGCCCTGGTGACC-3´). Samples were measured in triplicates.

#### Determination of proinflammatory cytokines

To detect proinflammatory cytokines, the culture medium was replaced with serum-free medium for 24 h before collecting the conditioned culture medium. Human Inflammation 11-Plex Panel: IFNγ, IL-1α, IL-1β, IL-6, IL-8, IL-10, IL-12p70, IL-27, IP-10, MCP-1, and TNFα (AimPlex Biosciences, Inc.) was used to determine cytokines using a BD FACSVerseTM flow cytometer (BD Biosciences, Franklin Lakes, NJ, USA) according to the manufacturer’s protocol. Data were analyzed using FlowJo 10 software (FlowJo LLC, Ashland, OR, USA).

#### Data processing and statistical analysis

Graphs were generated using GraphPad Prism 5.04 (GraphPad Software, La Jolla, CA, USA). The data are expressed as the mean ± SEM from three independent experiments. Statistical differences for two groups were analyzed by Student’s *t*-test, ^****^p < 0.0001; ^***^p < 0.001; ^**^p < 0.01; *p < 0.05. Time-lapse microscopy data were analyzed by the Incucyte SX1 platform. Quantitative analysis of immunoblots was done by Image J 1.48v program with GAPDH as a loading control. Migration of glioma cells was analyzed using Python 3.9.18 [47], with statistical analysis performed via SciPy 1.11.4 [48] and visualizations generated using Seaborn 0.13.0 [49].

### In vivo experiments

#### Experimental animals (median lethal dose experiment)

This study involved male and female Swiss mice aged 8 to 10 weeks weighing 24 to 28 g, respectively (Veterinary Service Center, Military Health Care Department, Belgrade, Serbia). Plastic cages (Macrolon^®^ cage type 4, Bioscape, Germany) with sawdust bedding (Versele-Laga, Belgium) certificated as having contaminant levels below toxic concentrations were used for animal housing. Environmental conditions were carefully maintained using a central computer-assisted system, maintaining a temperature of 22 ± 2 °C, relative humidity of 55 ± 15%, 15–20 air changes/hour, and artificial lighting of approximately 220 lx (12 h light/dark cycle). The mice had ad libitum access to Gebi commercial pellets for mice (from Čantavir, Serbia) and tap water filtered through a 1.0 *µ*m filter (Skala Green, Serbia), sourced from municipal mains.

All animal care and experimental procedures were approved by the Ethical Commission for the Protection of Animal Welfare, Faculty of Veterinary Medicine, University of Belgrade, Serbia (opinion 12/2021, decision No.: 01-442/1 from 27.05.2021), which was confirmed by the Ethical Committee of the Veterinary Directorate, Ministry of Agriculture, Forestry and Water Management, Belgrade, Serbia (decision No. 323-07-06024/2021-05 from 10.06.2021).

#### Determination of intraperitoneal median lethal dose of 5 and 7 in mice

The median lethal dose (LD_50_) of compounds 5 and 7 was determined using four groups of male and female mice, respectively, with each experimental group consisting of 8 animals. Increasing doses of each compound were administered intraperitoneally (*i.p*.) to separate groups of mice of both genders, as detailed in Table S1 (SI). Intraperitoneal injection of compounds 5 and 7 was performed in the lower right quadrant of the abdomen, directed towards the head at a 30–40° angle horizontally to prevent damage to the urinary bladder, cecum, and other abdominal organs. To assess dose-dependent acute toxicity, mice of both genders were divided into four dose groups: a low-dose group (producing no compound-related toxicity), a mid-dose group (eliciting minimal signs of compound-induced toxicity), a high-dose group (resulting in prominent compound-induced toxic effects), and a highest-dose group (causing mortality in all treated animals) [32, 50, 51]. Namely, the current study was performed according to the OECD Guidelines in a GLP-compliant way [28–30].

#### The general condition of the experimental animals

After treatment, all animals were observed for toxic symptoms for 1, 2, 4, 6, 24 h, and seven days. Then, their general condition was observed daily throughout the experiment, lasting seven days.

#### Median lethal dose

After 24 h, all dead animals were counted, and the median lethal dose (LD_50_) for 5 and 7 was calculated according to the method of Litchfield & Wilcoxon [52].

## Supplementary Information

Below is the link to the electronic supplementary material.

Supplementary Material 1.

## Data Availability

The data that support the findings of this study are available from the corresponding authors upon reasonable request.

## Abbreviations

ADME: Absorption
ATP: Adenosine triphosphate
BBB: Blood-brain barrier
BJ: Normal human skin fibroblasts
CNS MPO: Central nervous system multiparameter optimization
CV: Crystal violet
DIS: Docetaxel-induced senescence
DMEM: Dulbecco´s modified Eagle´s medium
DTX: Docetaxel
FACS: Fluorescence-activated cell sorting
FBD: Fragment-based design
FBS: Fetal bovine serum
FKBP12: FK506 binding protein 12
FRB domain: FKBP12-rapamycin binding domain
HBD: Hydrogen-bond donor
HESI: Heated electro-spray ionization
HRMS: High-resolution mass spectra
HRP: Horseradish peroxidase
i.p.: Intraperitoneal
IR: Ionizing radiation
LD_50_: Median lethal dose
logD: Distribution coefficient at pH 7.4
logP: Partition coefficient
mTOR: Mechanistic target of rapamycin
mTORC1: mTOR complex 1
mTORC2: mTOR complex 2
Mw: Molecular weight
NMR: Nuclear magnetic resonance
p16: p16INK4A
p21: p21waf1
Pd(dppf)Cl_2_.DCM: [1,1’-Bis(diphenylphosphino)ferrocene]palladium(II) dichloride in complex with dichloromethane
pKa: Acid-base dissociation constant
RPE-1: Human non-transformed telomerase-immortalized retinal pigment epithelial cells
S6K: p70 S6 kinase
SASP: Senescence-associated secretory phenotype
Tem: Temsirolimus
TLC: Thin-layer chromatography
TMZ: Temozolomide
tPSA: Topological polar surface area
TR-FRET: Time-resolved fluorescence resonance energy transfer
