# Supplementary material for: In silico screening, synthesis, and biological evaluation of pyrazolopyrimidine-derived mTOR inhibitors for anticancer and senomorphic effects
Source: Cancer Cell Int. 2026 May 6;26:247. doi: 10.1186/s12935-026-04308-0 (PMC13321562; doi:10.1186/s12935-026-04308-0)
Supplement: Supplementary file 1 — Supplementary Material 1. [file 12935_2026_4308_MOESM1_ESM.docx]

**Supporting Information**

David Rysanek^1*#^, Zofia Chrienova^2*^, Dorota Stary^3,4^, Marek Bajda^3^, Josef Novak^1^, Pavla Vasicova^1^, Jirina Kroupova^1^, Rudolf Andrys^2^, Adam Skarka^2^, Erika Rousarova^5^, Jan Capek^6^, Tomas Rousar^6^, Zoran Milovanovic^7^, Jelica Grujic-Milanovic^8^, Vesna Jacevic^2,9,10^, Jayaprakash N. Kolla^11^, Martin Popr^11^, Dominika Maurencova^1^, Patrik Oleksak^2^, Lukas Fresser^2^, Kamil Kuca^12^, Zdenek Hodny^1^ and Eugenie Nepovimova^2#^

*^1^Laboratory of Genome Integrity, Institute of Molecular Genetics of the Czech Academy of Sciences, Videnska 1083, 142 20 Prague, Czech Republic.*

*^2^Department of Chemistry, Faculty of Science, University of Hradec Kralove, Rokitanskeho 62, 500 03 Hradec Kralove, Czech Republic.*

*^3^Department of Physicochemical Drug Analysis, Faculty of Pharmacy, Jagiellonian University Medical College, Medyczna 9, 30-688 Cracow, Poland.*

*^4^Doctoral School of Medical and Health Sciences, Jagiellonian University Medical College, Medyczna 9, 30-688 Cracow, Poland.*

*^5^Department of Analytical Chemistry, Faculty of Chemical Technology, University of Pardubice, Studentska 573, 532 10, Pardubice, Czech Republic.*

*^6^Department of Biological and Biochemical Sciences, Faculty of Chemical Technology, University of Pardubice, Studentska 573, 532 10, Pardubice, Czech Republic.*

*^7^Special Police Unit, Ministry of Interior, Trebevićka 12/A, 11030 Belgrade, Serbia.*

*^8^University of Belgrade – Institute for Medical Research, National Institute of the Republic of Serbia, Department for Cardiovascular Research, Dr Subotića 4, 11029 Belgrade, Serbia.*

*^9^Department of Experimental Toxicology and Pharmacology, National Poison Control Centre, Military Medical Academy, 11 Crnotravska, 11040 Belgrade, Serbia.*

*^10^Medical Faculty of the Military Medical Academy, University of Defence, 11 Crnotravska, 11040 Belgrade, Serbia.*

*^11^CZ-OPENSCREEN: National Infrastructure for Chemical Biology, Institute of Molecular Genetics of the Czech Academy of Sciences, Videnska 1083, 14220 Prague 4, Czech Republic.*

*^12^Centre for Basic and Applied Research, Faculty of Informatics and Management, University of Hradec Kralove, Hradec Kralove 500 03, Czech Republic.*

^*^These authors contributed equally to this work and share first authorship.

^#^corresponding authors: david.rysanek@img.cas.cz; eugenie.nepovimova@uhk.cz


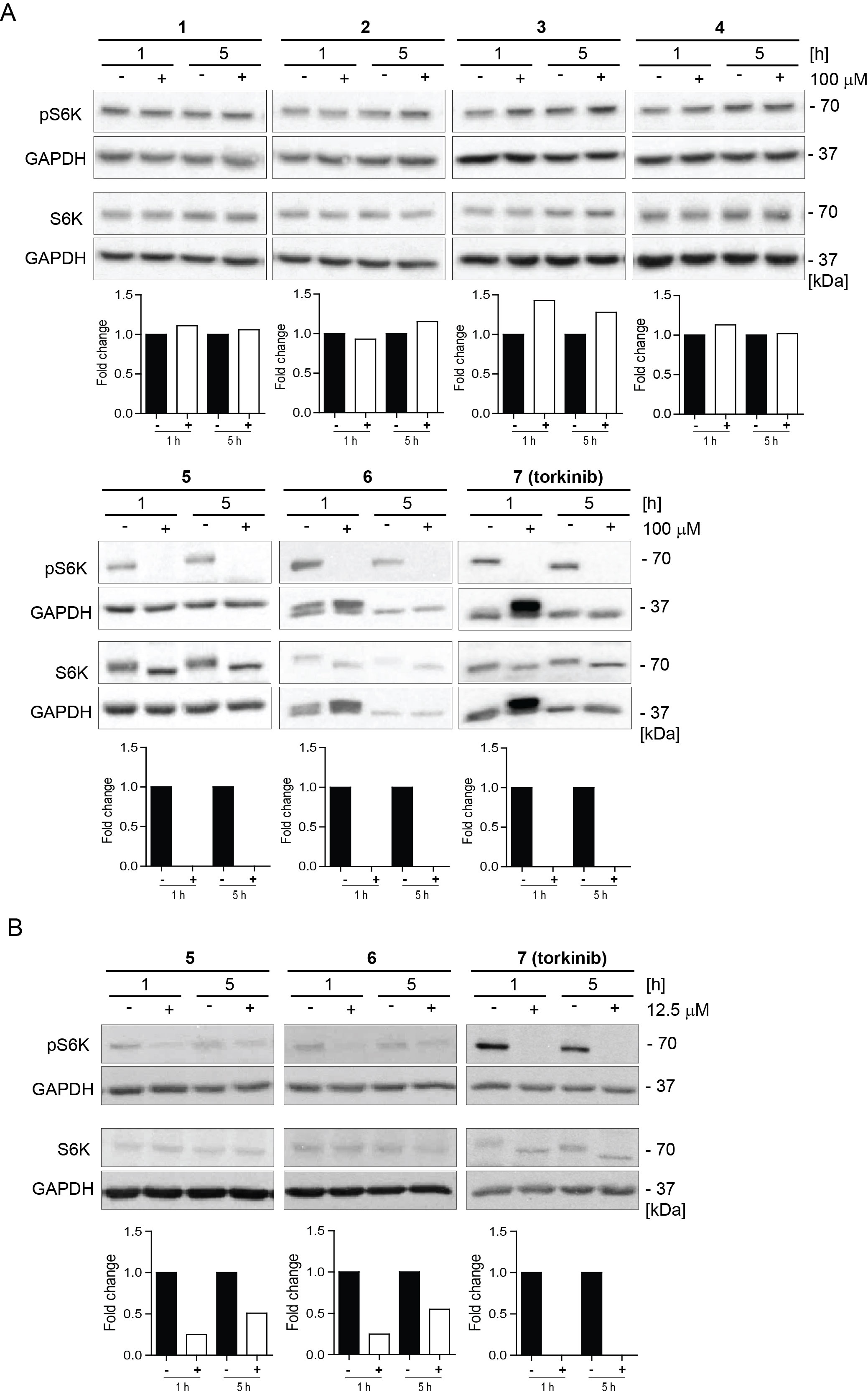


**Figure S1**. Detection of mTORC1 kinase inhibition with compounds **1** – **7**. (A) BJ cells were exposed to compounds **1** – **7** (100 *μ*M) for 1 and 5 h. (B) BJ cells were exposed to a 12.5 *μ*M concentration of compounds **5**, **6**, and **7** for 1 and 5 h. The levels of p70 S6 kinase (S6K) threonine 389 (Thr389) phosphorylation, mediated by mTORC1, and S6K total level were detected by immunoblotting using specific antibodies. GAPDH was used as a loading control. Quantitative analysis of immunoblots was performed using the ImageJ 1.48v program, with GAPDH as the internal control.


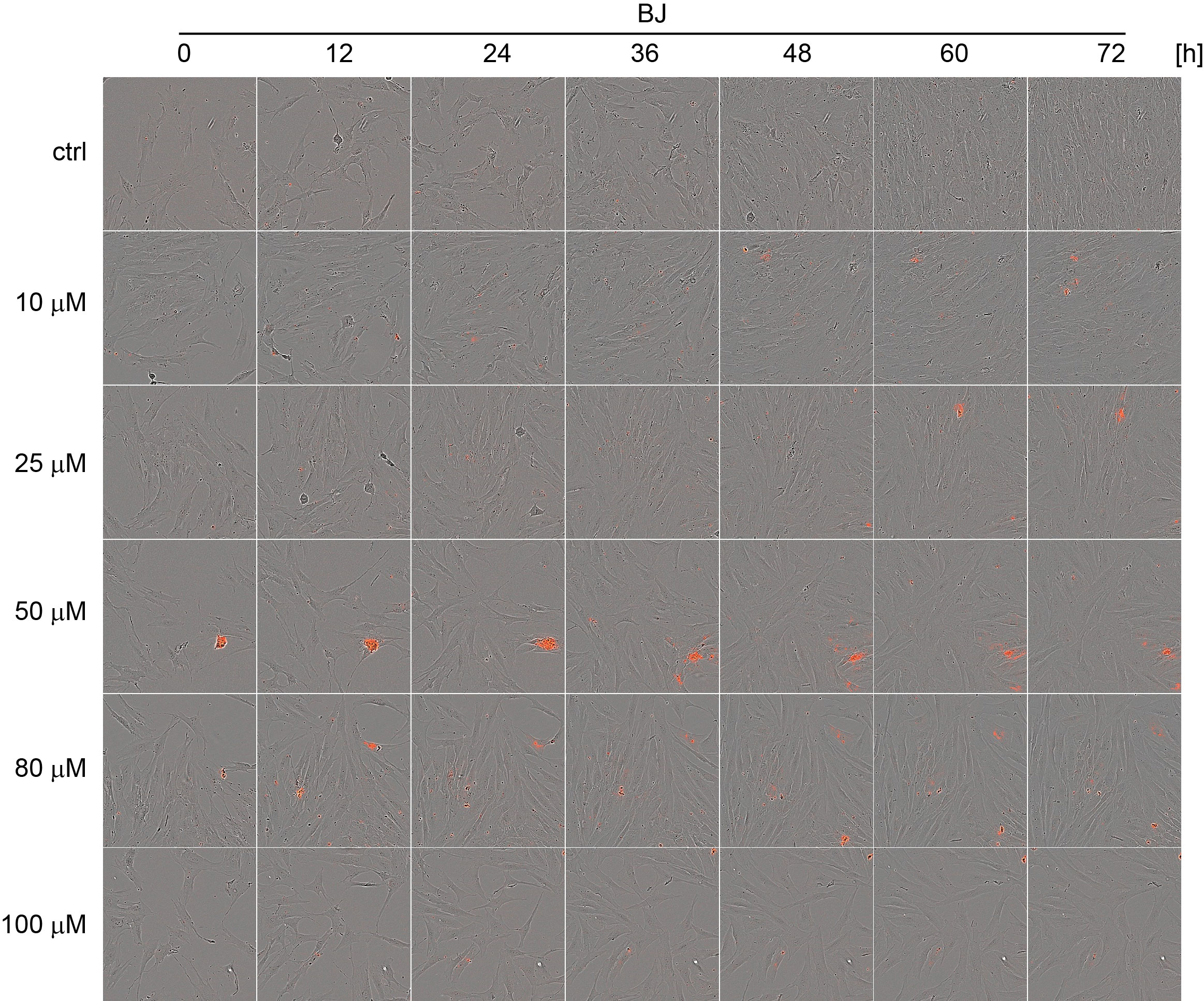


**Figure S2A**


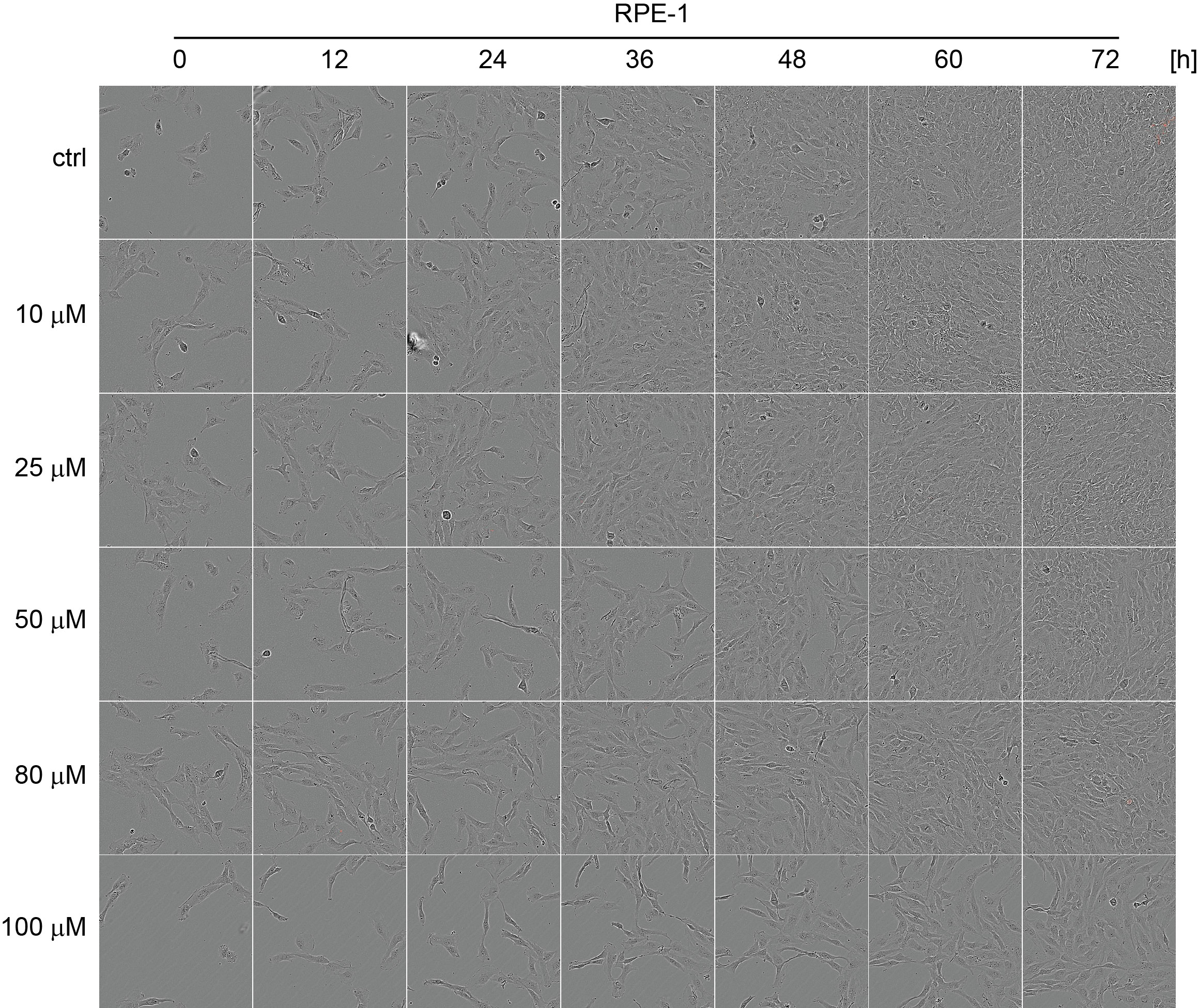


**Figure S2B**


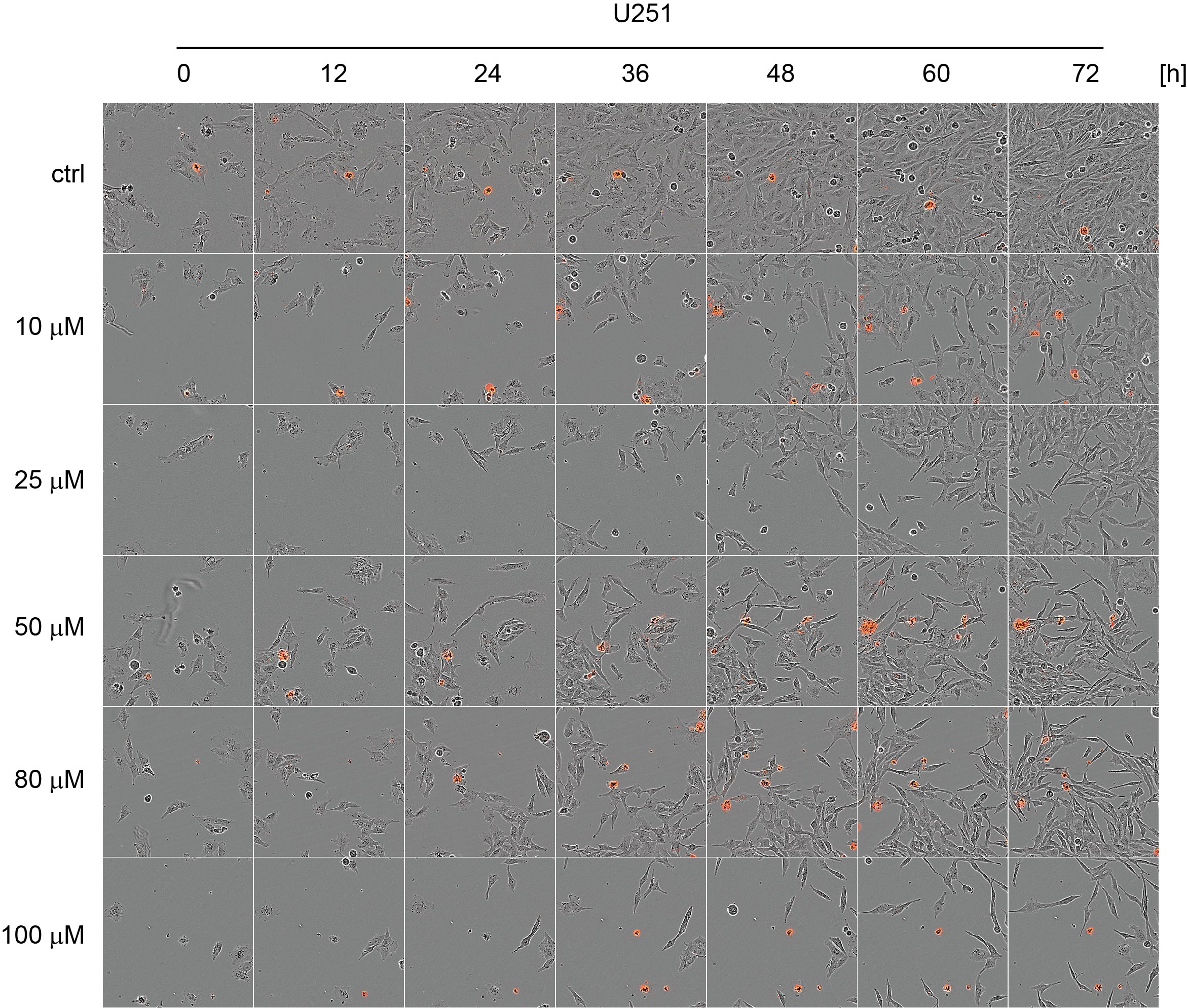


**Figure S2C**


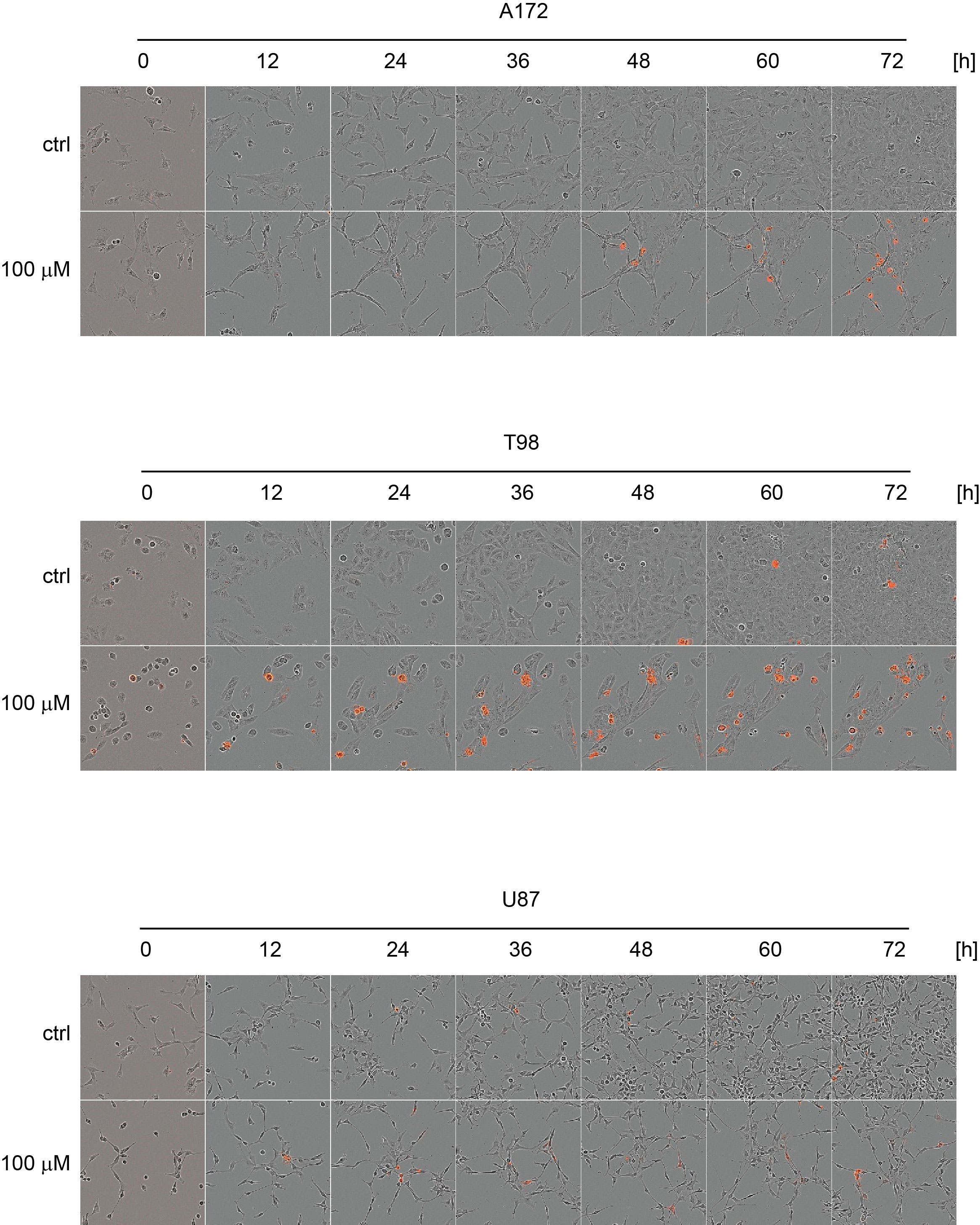


**Figure S2D**


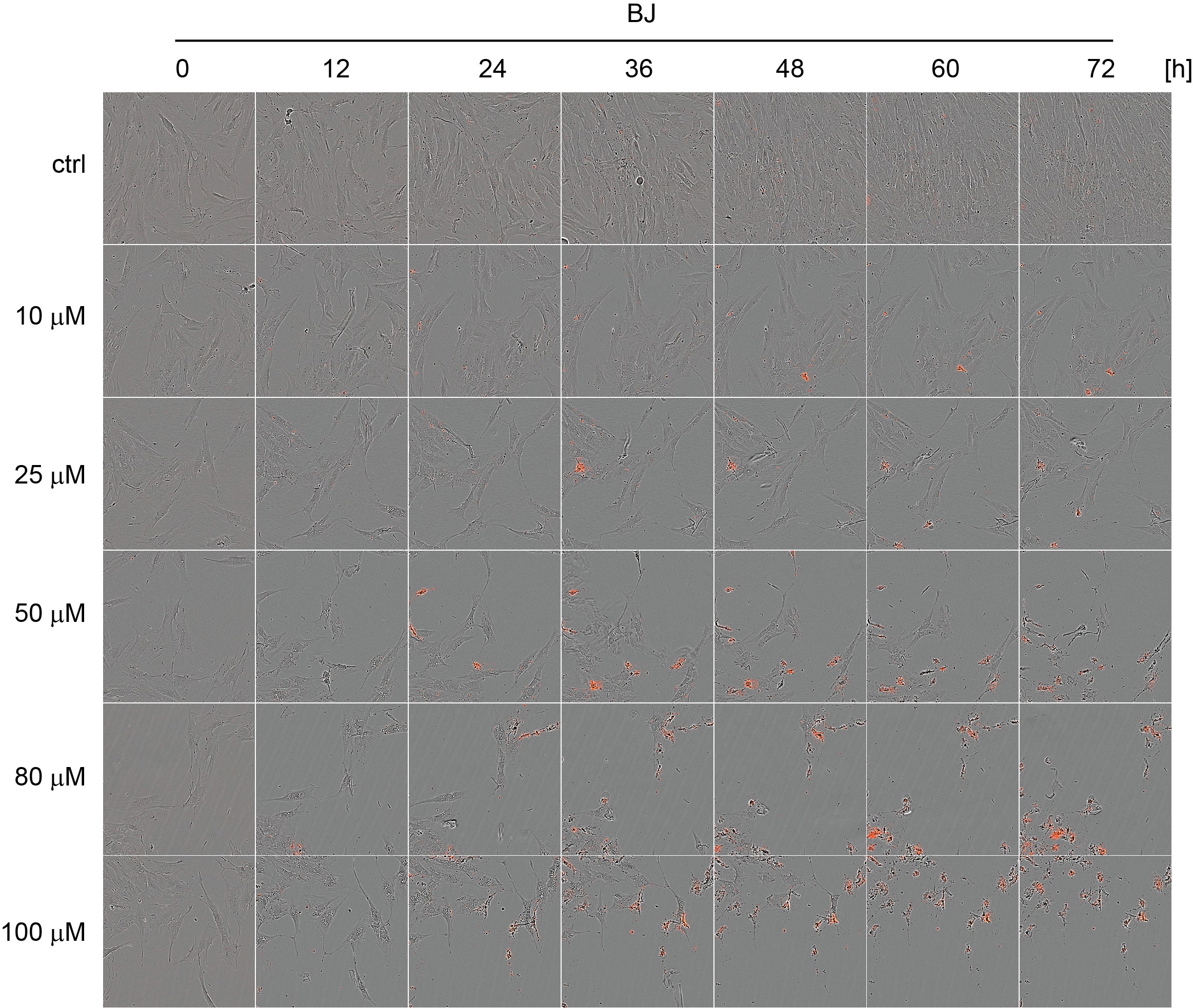


**Figure S2E**


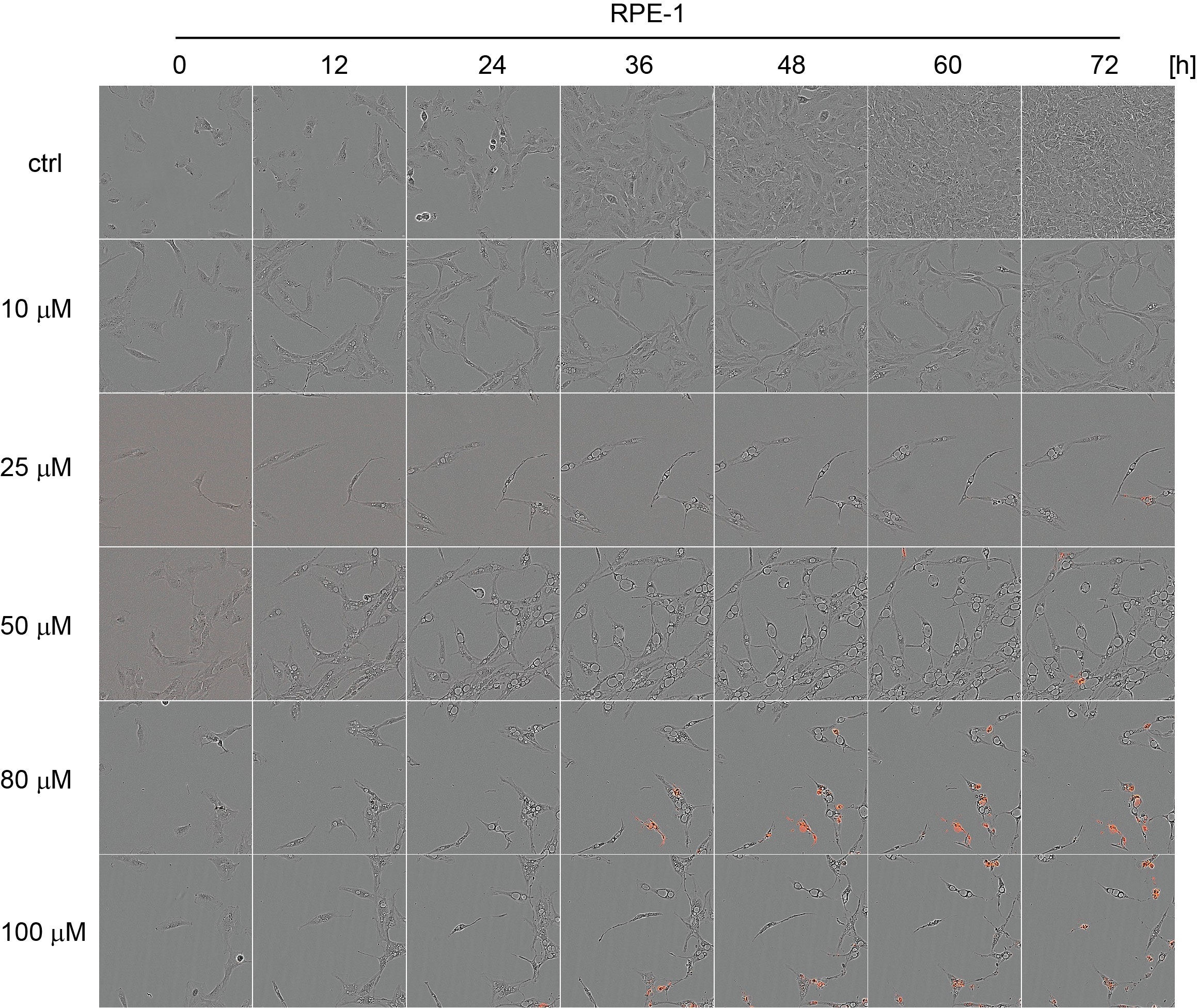


**Figure S2F**


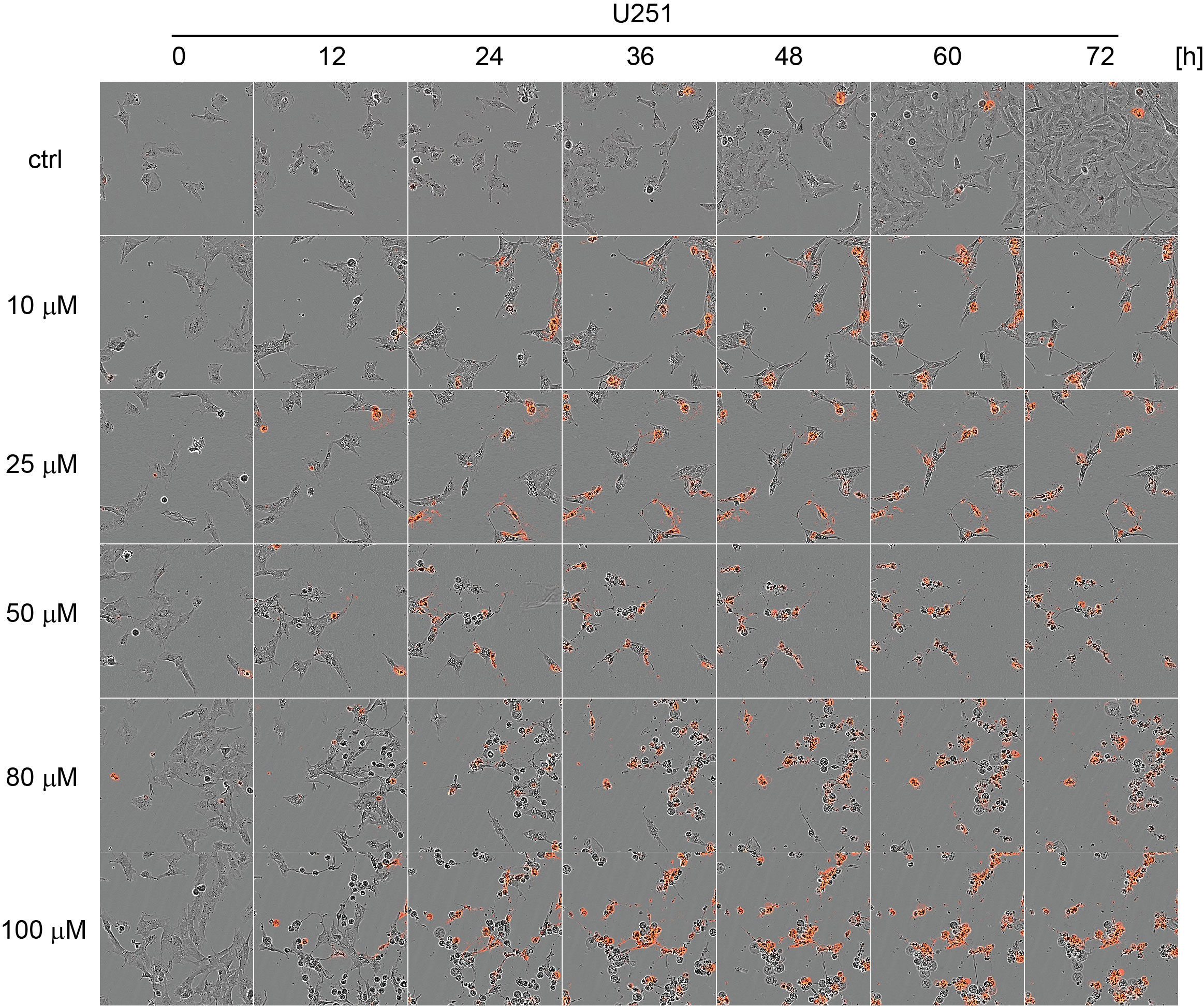


**Figure S2G**


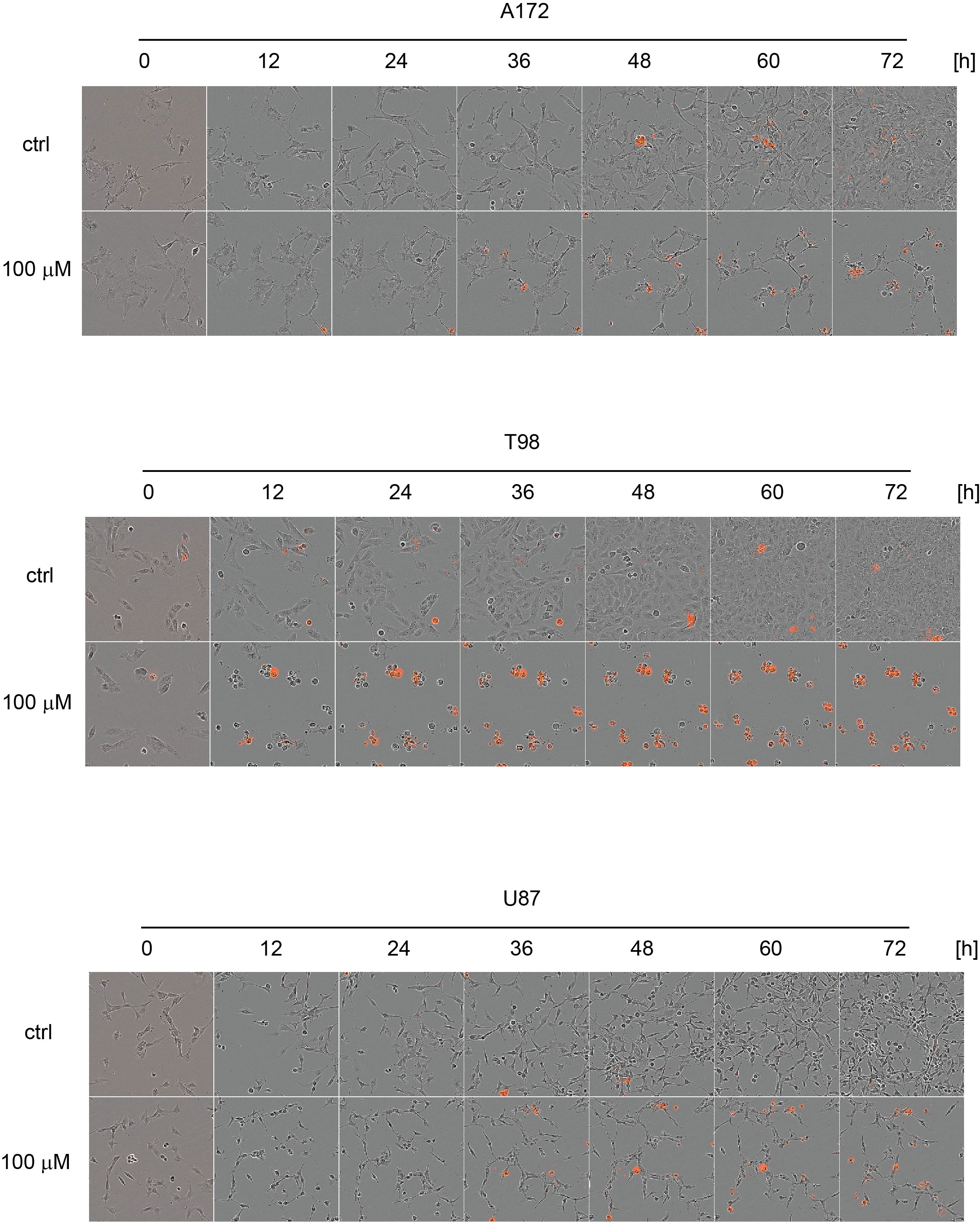


**Figure S2H**


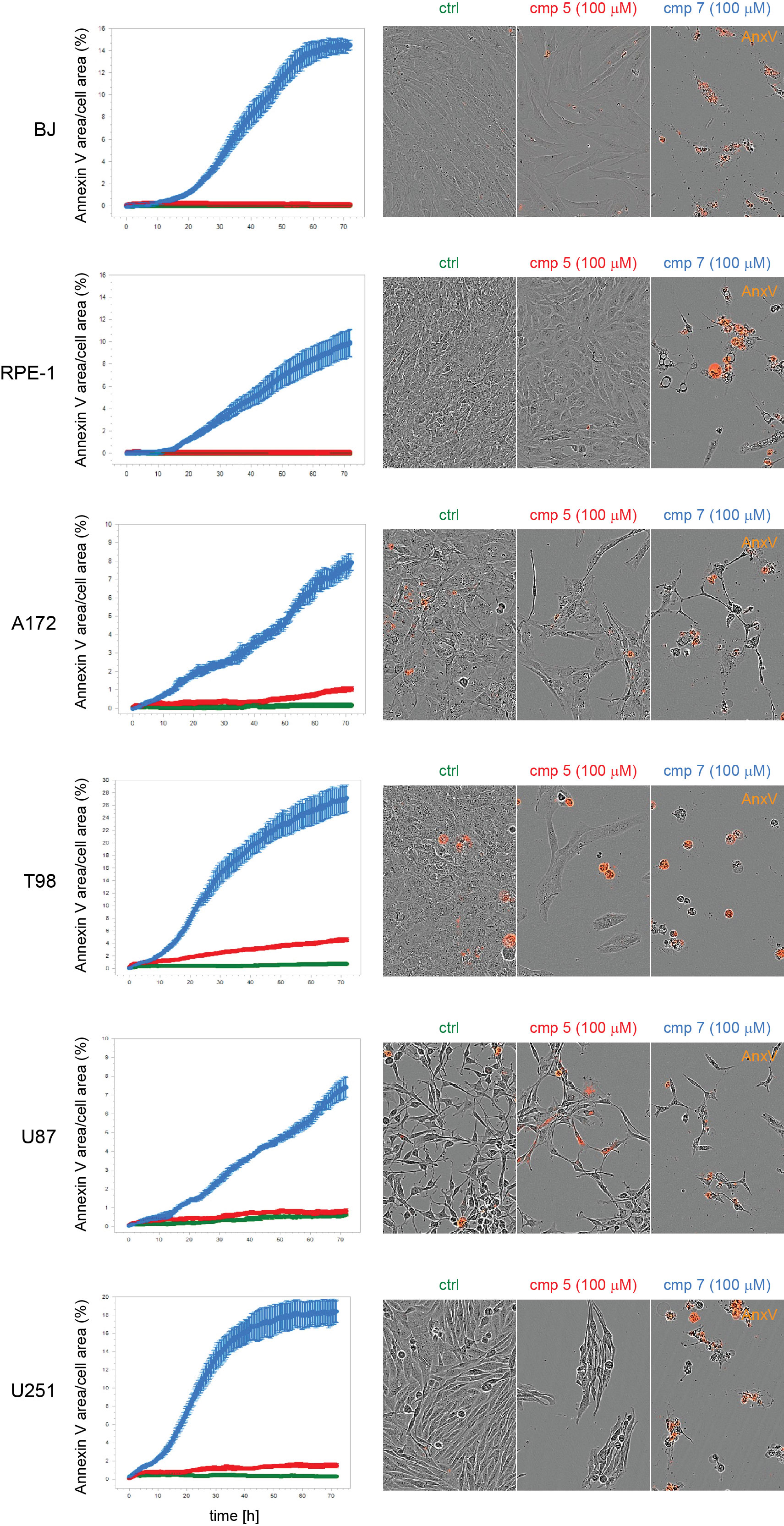


**Figure S2I**

**Figure S2**. Cytostatic and cytotoxic effects induced by compounds **5** in proliferating (A) BJ, (B) RPE-1, (C) U251, (D) A172, T98, and U87 cells and compound **7** in proliferating (E) BJ, (F) RPE-1, (G) U251, (H) A172, T98, and U87 cells. The cytotoxic effect of compounds **5** and **7** (10 – 100 *μ*M) was demonstrated by time-lapse microscopy (0 – 72 h) using the Incucyte SX1 platform. Representative 100 × 100 μm regions of images captured at 0, 12, 24, 36, 48, 60, and 72 h are presented. The red colour represents annexin V staining of apoptotic cells. (I) The graphs show changes in cell death as detected by fluorescently labelled annexin V staining. Average annexin V staining normalized to cell confluence, with standard error from four images, is shown. A representative 310 × 450 μm region of endpoint images (72 h) is shown.


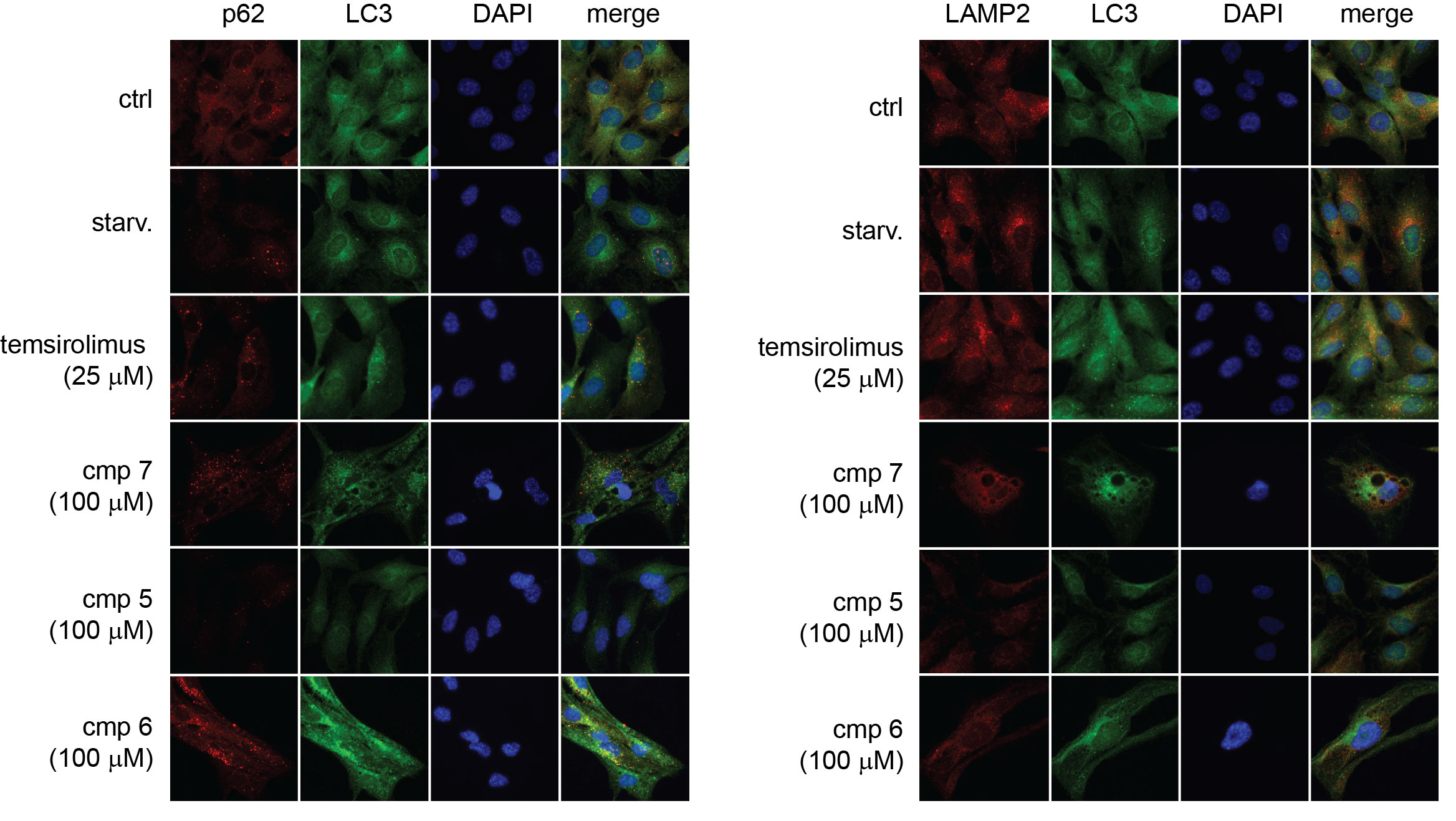


**Figure S3A**


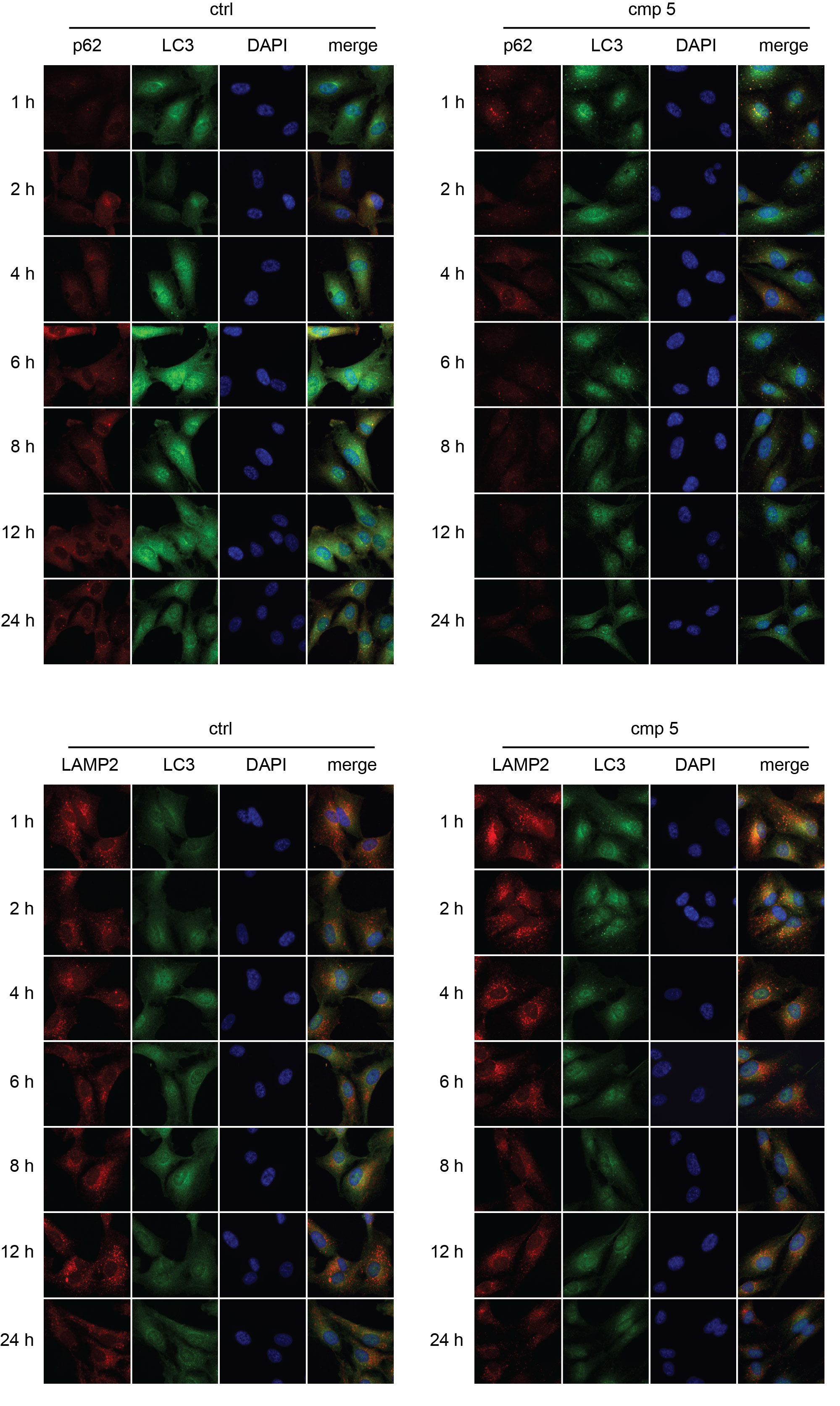


**Figure S3B**


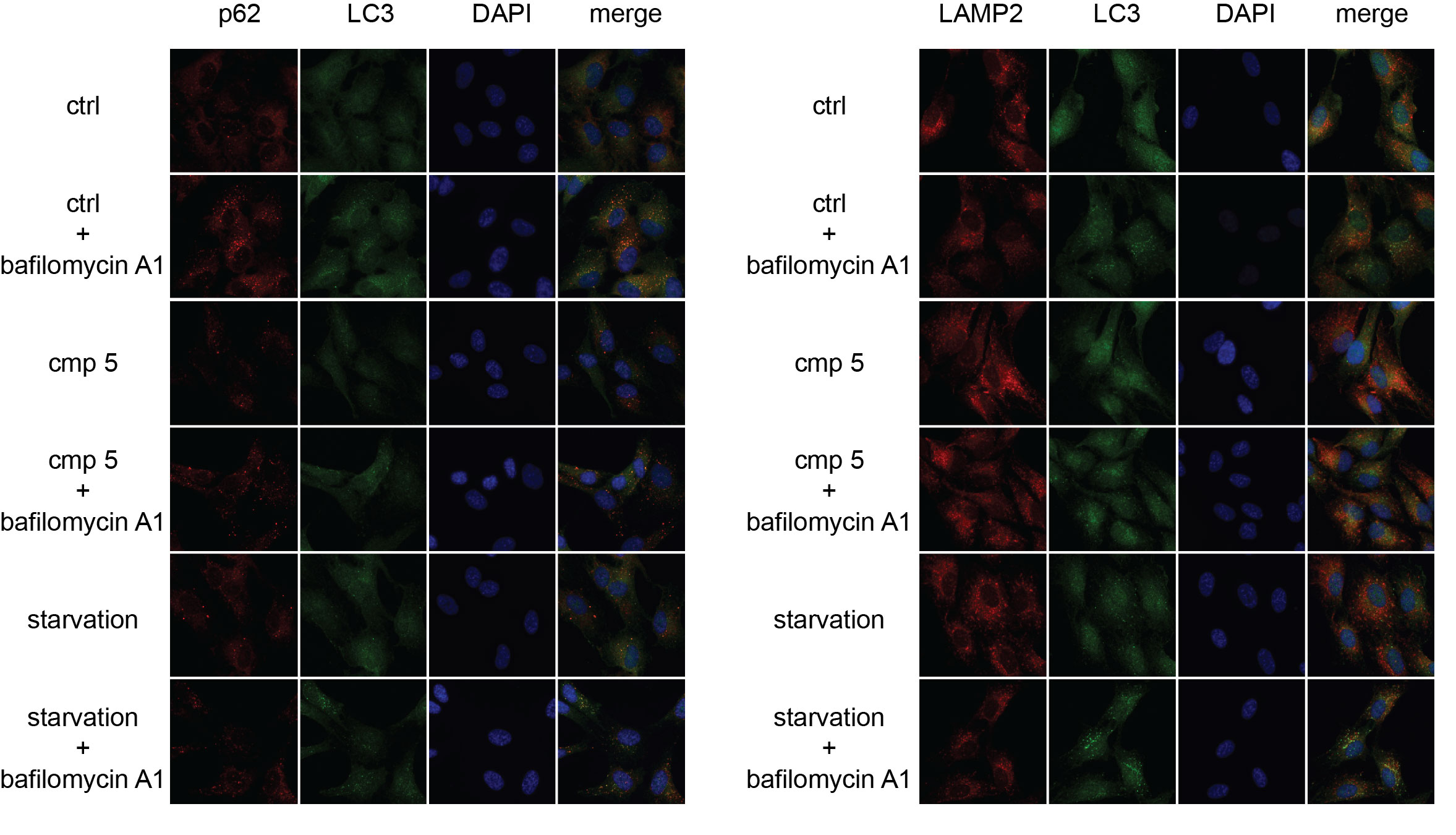


**Figure S3C**


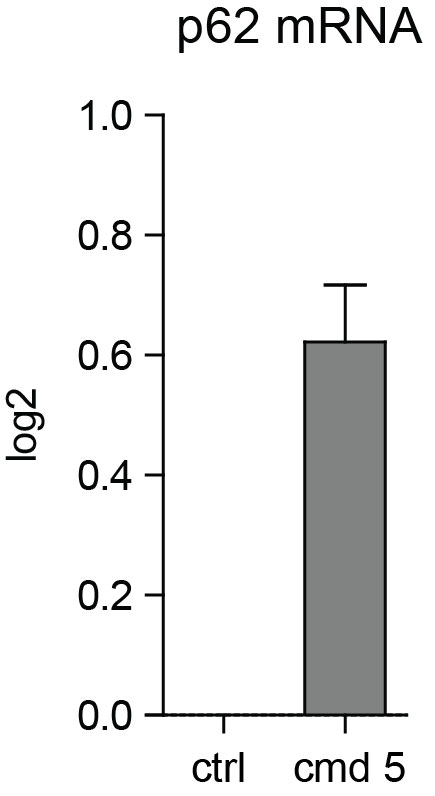


**Figure S3D**

**Figure S3**. Detection of autophagy. (A) RPE-1 cells were cultivated in medium without serum (starvation) with compounds **5**, **6**, and **7** (100 µM), and temsirolimus (25 µM) for 24 h. Cells were stained with LC3, p62, DAPI, and LC3, LAMP2, DAPI. (B) Compound **5** was added at 1, 2, 4, 6, 8, 12, and 24 h, and cells were stained with LC3, p62, DAPI and LC3, LAMP2, DAPI. (C) To detect autophagy flux, bafilomycin A1 was used for 1 h (100 nM) on cells cultivated for 23 h in full medium (ctrl), in medium withour serum (starvation), and with compound **5**. Cells were stained with LC3, p62, DAPI, and LC3, LAMP2, and DAPI. Representative 100 × 100 μm regions from captured images are shown. (D) p62 mRNA level determined by real-time reverse transcription-polymerase chain reaction in RPE-1 cells exposed to compound **5** (100 µM) for 24 h.


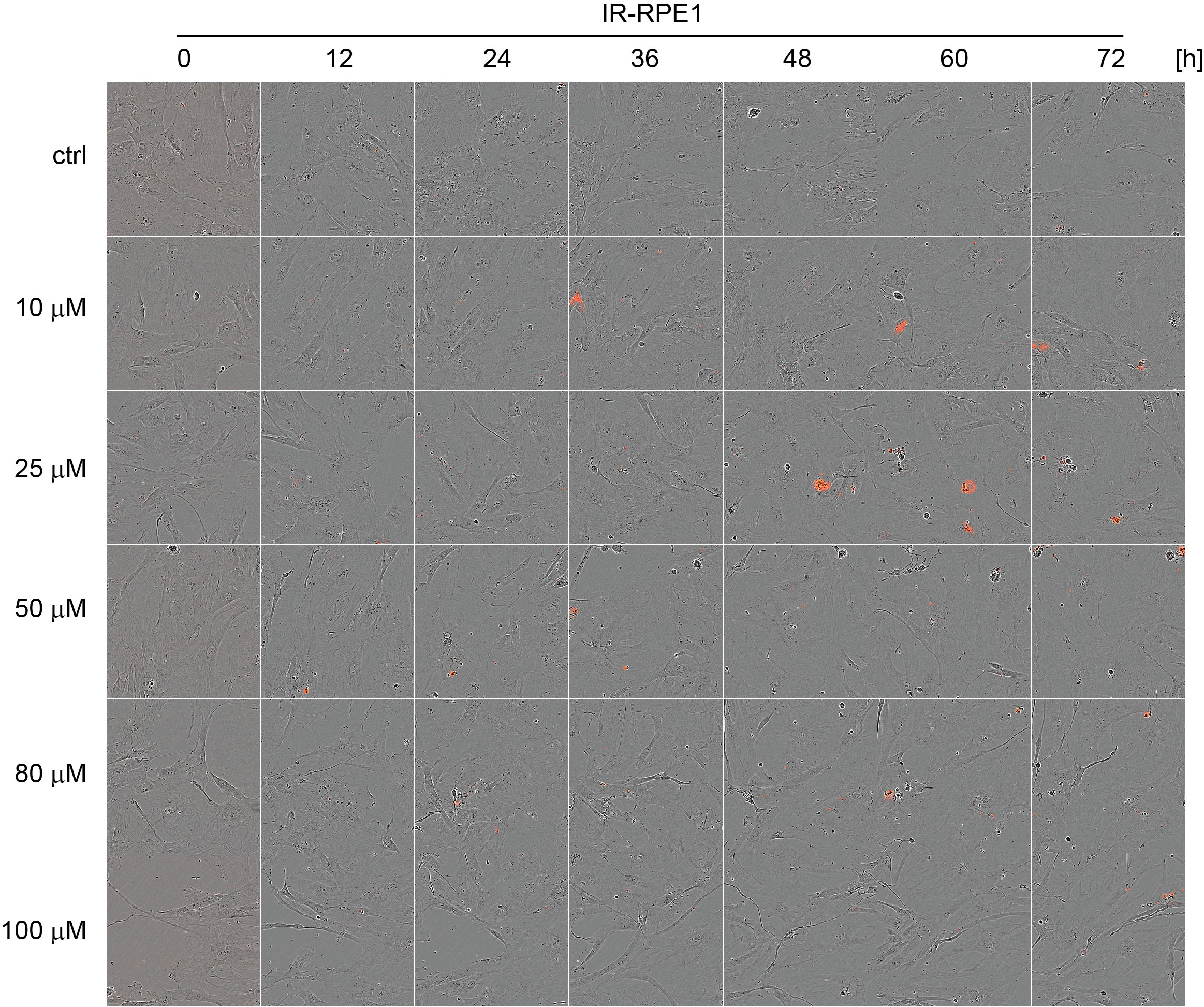


**Figure S4A**


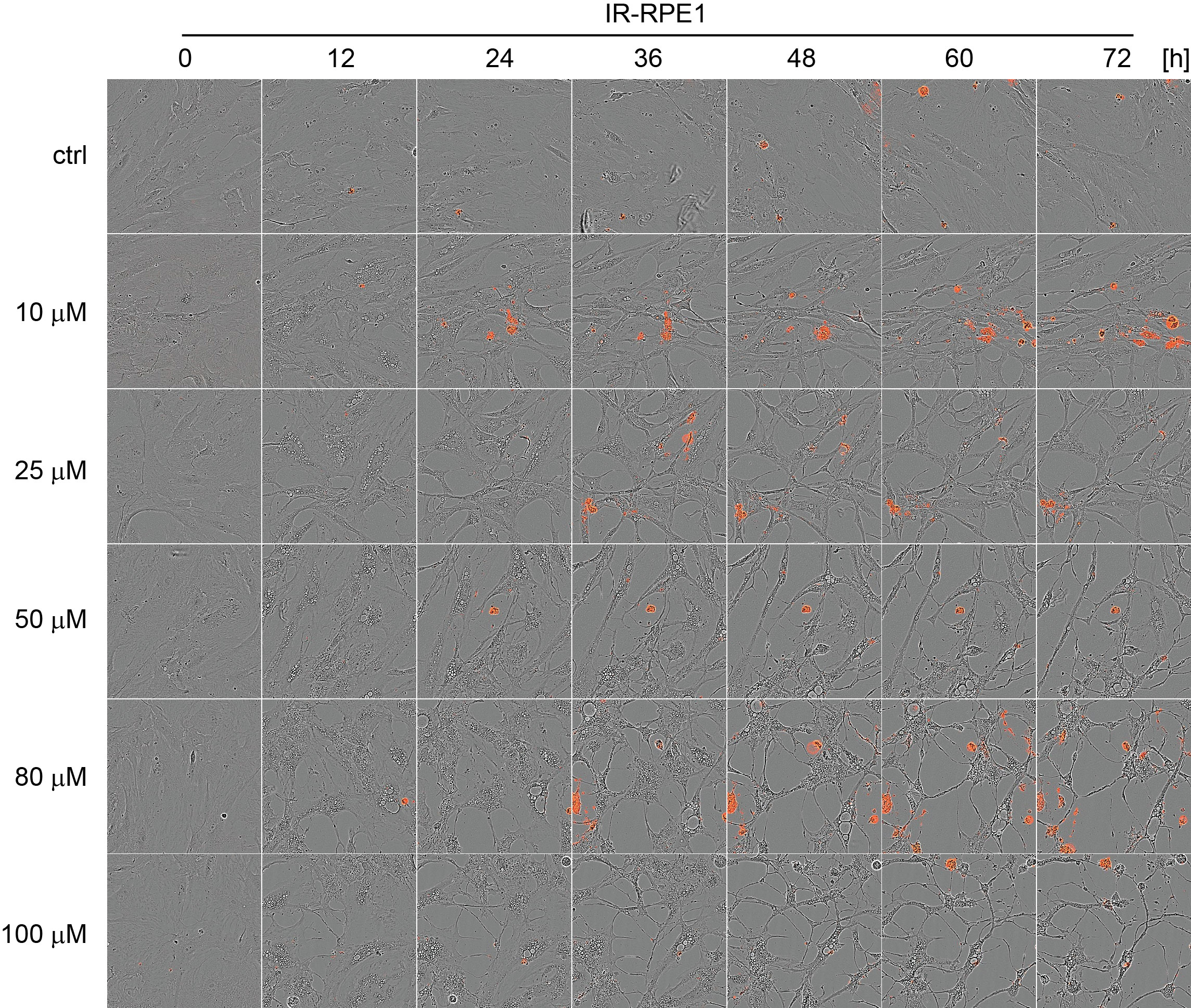


**Figure S4B**

**
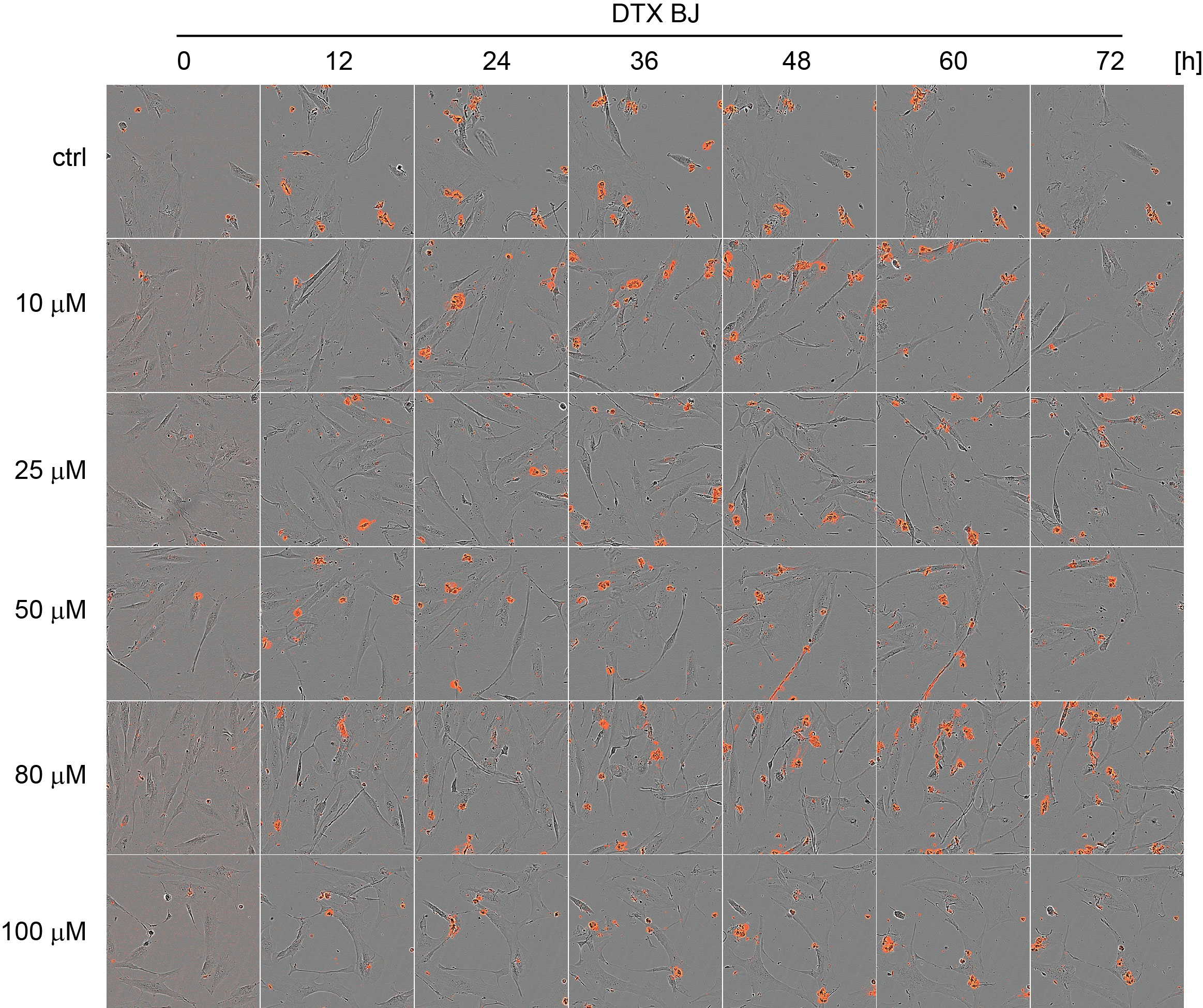
**

**Figure S4C.**

**
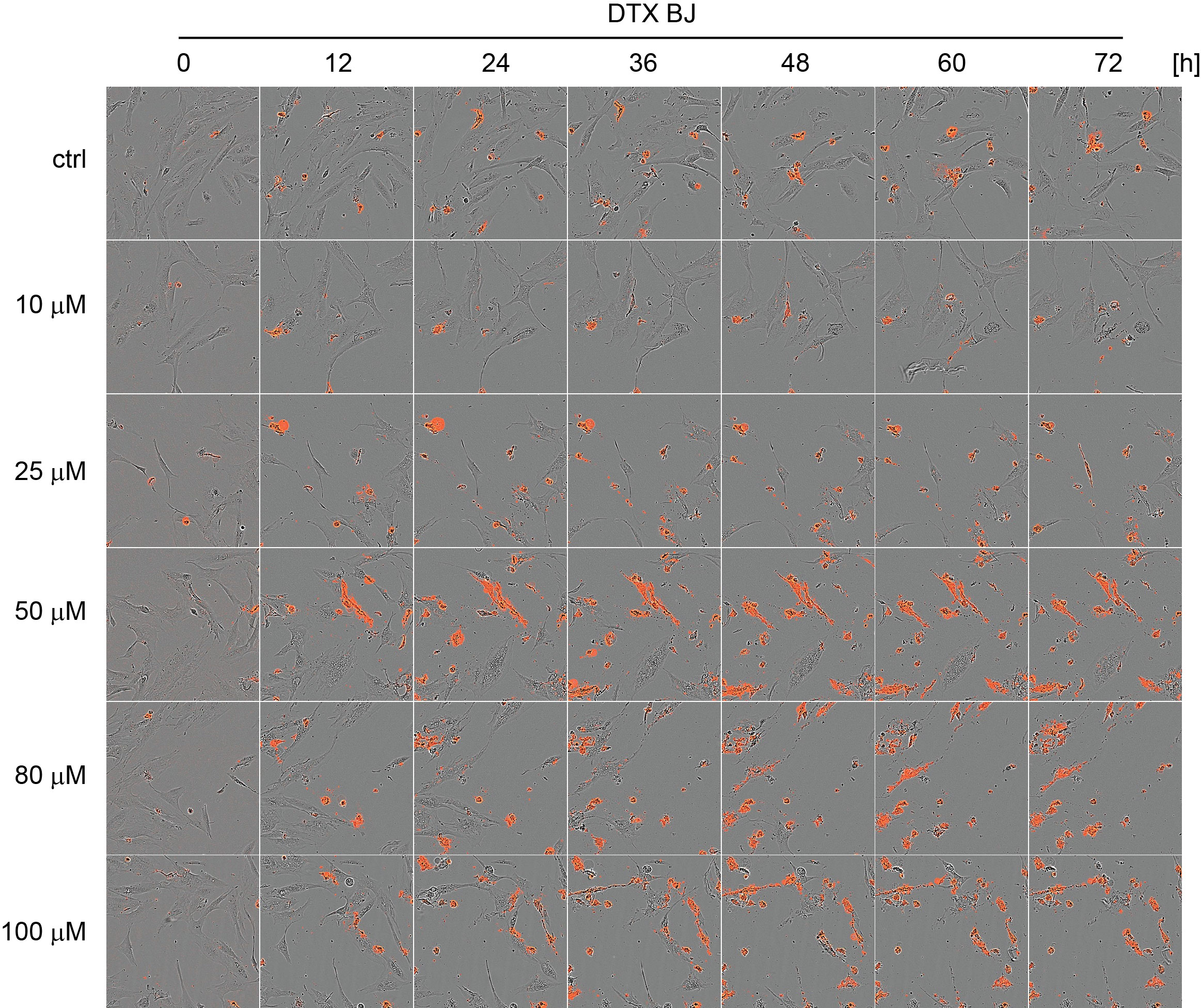
**

**Figure S4D.**

**
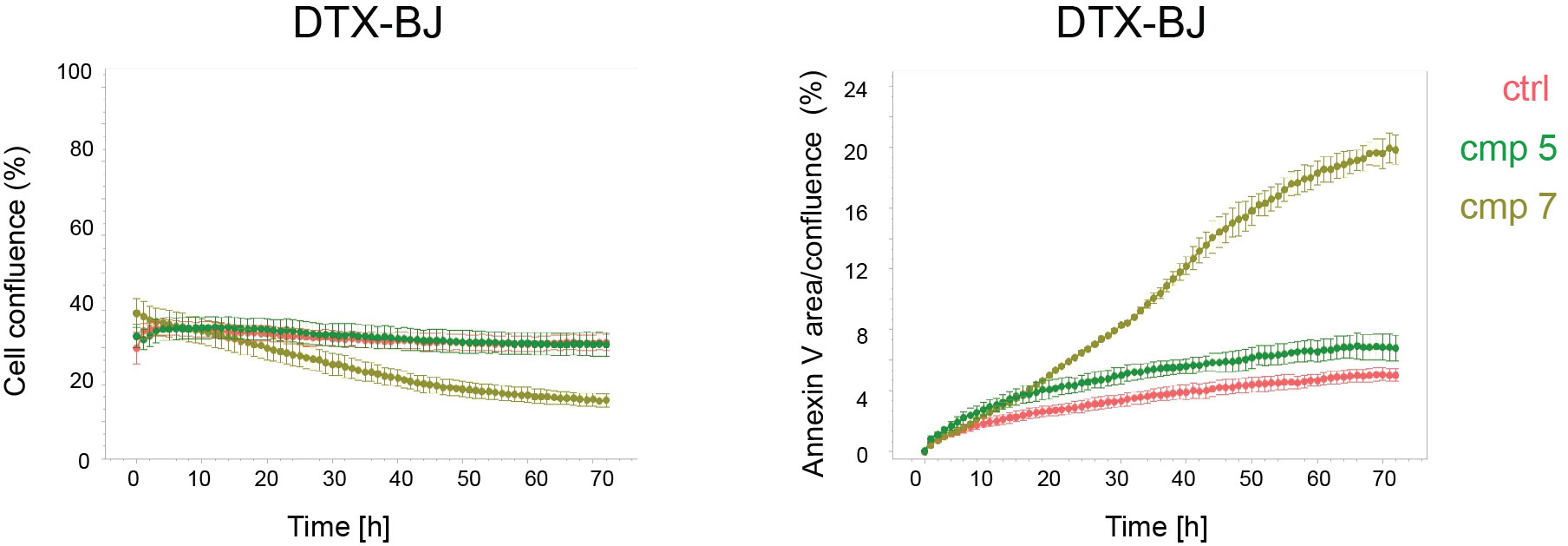
**

**Figure S4E.**

**
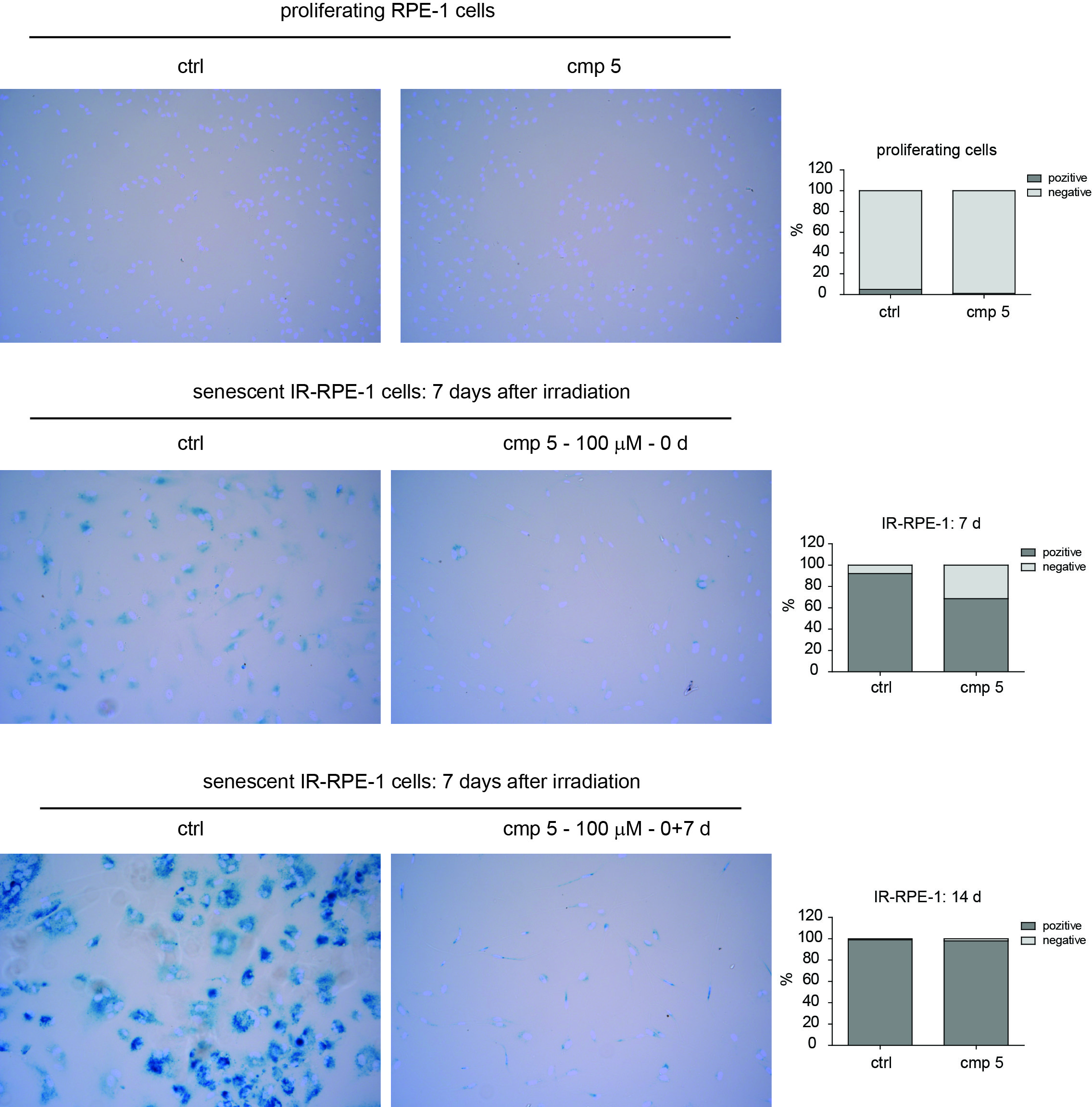
**

**Figure S4F.**

**Figure S4**. Cytotoxic effect in senescent IR-RPE-1 cells induced by compounds **5** (A) and compound **7** (B) and in senescent DTX-BJ cells induced by compounds **5** (C) and compound **7** (D) in the concentration range of 10 – 100 *μ*M, demonstrated by time-lapse microscopy (0 – 72 h) using the Incucyte SX1 platform. (E) The graphs plot changes in cell confluence and cell death. Average cell confluence and annexin V staining positive area normalized to cell confluence with standard error from four images are shown. Representative 400 x 400 μm regions of images captured at 0, 12, 24, 36, 48, 60, and 72 h are presented. The red colour represents annexin V staining of apoptotic cells. (F) Confirmation of cellular senescence for the experiment shown in Figure 11 using the detection of senescence-associated beta-galactosidase activity.

**Supplementary Table 1.** The selection of dose levels for acute toxicity study of compounds **5** and **7** in mice.

| Tested compound (mg/kg *i.p.*) | Mice (male/female) |
| --- | --- |
| **5** | 250.0 |
|  | 500.0 |
|  | 750.0 |
|  | 1000.0 |
| torkinib (**7**) | 500.0 |
|  | 750.0 |
|  | 1000.0 |
|  | 1250.0 |
